# Distinct conformations of the HIV-1 V3 loop crown are targetable for broad neutralization

**DOI:** 10.1038/s41467-021-27075-0

**Published:** 2021-11-18

**Authors:** Nikolas Friedrich, Emanuel Stiegeler, Matthias Glögl, Thomas Lemmin, Simon Hansen, Claus Kadelka, Yufan Wu, Patrick Ernst, Liridona Maliqi, Caio Foulkes, Mylène Morin, Mustafa Eroglu, Thomas Liechti, Branislav Ivan, Thomas Reinberg, Jonas V. Schaefer, Umut Karakus, Stephan Ursprung, Axel Mann, Peter Rusert, Roger D. Kouyos, John A. Robinson, Huldrych F. Günthard, Andreas Plückthun, Alexandra Trkola

**Affiliations:** 1 Institute of Medical Virology, University of Zurich (UZH), Zurich, Switzerland; 2 Department of Computer Science, ETH Zurich, Zurich, Switzerland; 3 Department of Biochemistry, University of Zurich (UZH), Zurich, Switzerland; 4 Department of Mathematics, Iowa State University, Ames, IA USA; 5 Department of Chemistry, University of Zurich (UZH), Zurich, Switzerland; 6 Division of Infectious Diseases and Hospital Epidemiology, University Hospital Zurich (USZ), Zurich, Switzerland; 7 Present Address: Roche Diagnostics GmbH, Nonnenwald 2, 82377 Penzberg, Deutschland; 8 Present Address: Euler Institute, Faculty of Biomedicine, Università della Svizzera italiana (USI), Lugano, Switzerland; 9 Present Address: NGM Bio, 333 Oysterpoint Blvd, South San Francisco, CA 94080 USA; 10 Present Address: Innovent Biologics Inc, 168 Dongping Street, Suzhou Industrial Park, 215123 China; 11 Present Address: Office Research and Teaching, Medical Faculty, University of Zurich, Zurich, Switzerland; 12 Present Address: BeiGene Switzerland GmbH, Aeschengraben 27, 4051 Basel, Switzerland; 13 Present Address: Janssen Vaccines AG, Rehhagstrasse 79, 3018 Bern, Switzerland; 14 Present Address: ImmunoTechnology Section, Vaccine Research Center, NIAID, NIH, Bethesda, MD USA; 15 Present Address: Laboratory Medicine, Division of Clinical Chemistry, University Hospital Basel, Basel, Switzerland; 16 Present Address: Novartis Institutes for BioMedical Research, Chemical Biology & Therapeutics (CBT), Novartis Pharma AG, Virchow 16, 4056 Basel, Switzerland; 17 Present Address: University of Cambridge School of Clinical Medicine, Department of Radiology, Cambridge, CB2 0QQ UK; 18 Present Address: Roche Innovation Center Zurich, Roche Pharmaceutical Research and Early Development (pRED), Wagistrasse 10, 8952 Schlieren, Switzerland

**Keywords:** Viral proteins, X-ray crystallography, Antibodies, Antivirals, HIV infections

## Abstract

The V3 loop of the HIV-1 envelope (Env) protein elicits a vigorous, but largely non-neutralizing antibody response directed to the V3-crown, whereas rare broadly neutralizing antibodies (bnAbs) target the V3-base. Challenging this view, we present V3-crown directed broadly neutralizing Designed Ankyrin Repeat Proteins (bnDs) matching the breadth of V3-base bnAbs. While most bnAbs target prefusion Env, V3-crown bnDs bind open Env conformations triggered by CD4 engagement. BnDs achieve breadth by focusing on highly conserved residues that are accessible in two distinct V3 conformations, one of which resembles CCR5-bound V3. We further show that these V3-crown conformations can, in principle, be attacked by antibodies. Supporting this conclusion, analysis of antibody binding activity in the Swiss 4.5 K HIV-1 cohort (n = 4,281) revealed a co-evolution of V3-crown reactivities and neutralization breadth. Our results indicate a role of V3-crown responses and its conformational preferences in bnAb development to be considered in preventive and therapeutic approaches.

## Abstract (web summary)

The V3-crown of the HIV-1 envelope protein largely elicits non-neutralizing antibodies. Here, the authors show that the V3-crown can be targeted by broadly neutralizing designed ankyrin repeat proteins recognizing two conformations one of which resembles CCR5- bound V3.

## Introduction

HIV-1 entry depends on the interaction of the variable loop 3 (V3) of its envelope (Env) protein with an HIV co-receptor, commonly CCR5 or CXCR4^1–3^. In line with its critical function in entry, the three sections of the V3 - (i) the base (residues 296–299 and 327–331 within the HxB2 reference strain), (ii) the stem (residues 300–303 and 321–326), and (iii) the crown (residues 304–320)^4,5^ - are largely conserved, making the V3 loop a potential prime target for neutralizing antibodies (nAbs), inhibitors and vaccine approaches. Yet, V3 is effectively shielded from antibody recognition by interaction with the variable loops 1 and 2 (V1V2)^6–10^. Conformational changes in the Env trimer upon CD4 receptor binding trigger a displacement of V1V2, lifting its trimer stabilizing function and enabling V3 to interact with the HIV co-receptors. The ensuing trimer opening exposes highly neutralization sensitive sites within the V3-crown^5,9,11,12^. However, the window of accessibility is normally not sufficient to allow V3-crown specific antibodies to effectively block infection. Prototypically, a vigorous V3 antibody response is elicited in almost all HIV-1 infected individuals, but bears little to no neutralization activity as it is mostly constituted of V3-crown Abs^9,13,14^.

Rare broadly neutralizing antibodies (bnAbs) targeting V3 overcome the access restriction on the trimer by binding to the conserved V3-base, involving the GDIR motif and surrounding glycans^15,16^. Considering the crucial function and conservation of the V3-crown, V3-crown inhibitors that can bypass V1V2 shielding could have immense therapeutic potential. If V1V2 shielding is artificially released, V3-crown Abs display extreme potency and breadth, thus confirming that accessibility, not specificity is the limiting factor^9^. Although a few cross-neutralizing V3-crown specific neutralizing Abs have been identified, overall they lack breadth compared to V3-base directed bnAbs^15–20^. The properties that distinguish cross-neutralizing V3-crown Abs from non-neutralizing V3 Abs are currently not fully understood. A capacity to recognize the V3-crown in distinct conformations has been proposed as a potential requirement^5,19,21–25^.

Using the Designed Ankyrin Repeat Proteins (DARPin) technology^26^, we previously developed the V3-crown specific DARPin 5m3_D12 that shares features with cross-neutralizing V3-crown Abs and has activity against difficult to neutralize (Tier-2) strains of HIV-1 subtype B^27^. Notably, 5m3_D12 partially by-passes V1V2 shielding, suggesting that generation of DARPin based broadly neutralizing V3-crown inhibitors may be possible. DARPins are based on a small rigid binding-protein scaffold, providing high target affinity and specificity for biomedical applications^26,28–30^. Due to their rigid binding surface complementary to folded protein domains, DARPins often bind in a conformation-specific manner^26,27,31^. Exemplary for this, 5m3_D12 displays a strong binding preference for a specific V3-crown conformation^27^. Identification of novel broadly neutralizing V3 DARPins may thus reveal V3 conformations that are relevant for infection. Building on the discovery of 5m3_D12, we here exploit the DARPin technology in order to define conformational states of the V3-crown that are targetable for broad neutralization.

## Results

### Broad neutralization via the V3-crown is possible

**Fig. 1:**
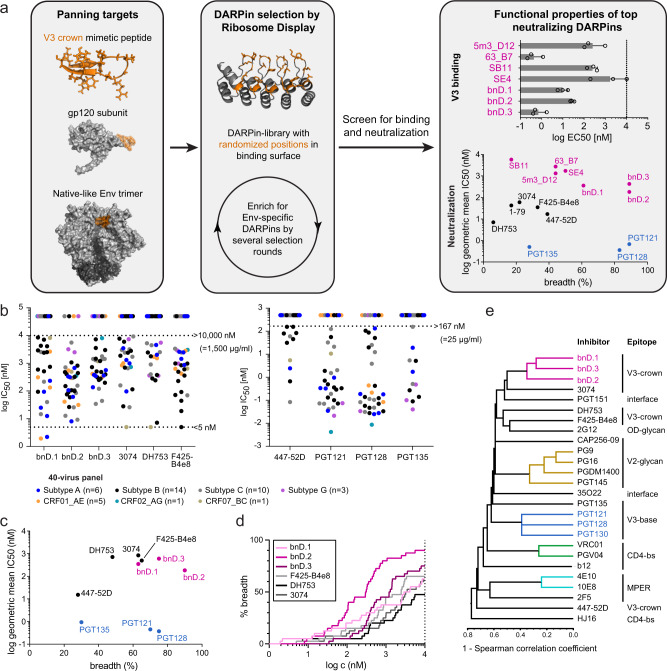
Selection of V3-crown reactive broadly neutralizing DARPins (bnDs). **a** Scheme of the selection workflow to identify V3-crown-reactive neutralizing DARPins. For details on panning, ribosome display selections, and the screening procedure, see Supplementary Fig. 1 and 2 as well as the Materials and Methods section. The right panel depicts V3-crown binding and neutralizing activity of the top six neutralizing DARPins and the reference DARPin 5m3_D12 selected for follow-up experiments (magenta). V3-crown nAbs (black) and V3-base bnAbs (blue) are shown for comparison. V3-crown binding is depicted as 50% effective concentration (EC_50_) (nM) derived by ELISA. Bars indicate the geometric mean EC_50_ values from three independent replicates (circles). Error bars depict the geometric standard deviation (SD). The neutralization breadth-potency plot is based on results from a multi-clade 18-virus panel (maximum concentrations probed: DARPins (10,000 nM or 20,000 nM) and mAbs (167 nM (= 25 μg/ml, commonly used for mAbs in this type of assay)) Supplementary Data 1). The geometric mean of 50% inhibitory concentration (IC_50_) values in **a** and **c** were calculated over all sensitive strains. **b** Extended neutralization analysis of bnDs and nAbs on a 40-virus panel (Supplementary Data 1). IC_50_ values (geometric mean of 1-5 independent replicates) are shown. Minimum (5 nM: only for mAbs 3074, DH753 and F425-B4e8) and maximum concentrations of DARPins (10,000 nM) and mAbs (10,000 nM or 167 nM (=25 μg/ml) probed are indicated by dashed lines. Viruses not neutralized are shown as dots above the dashed lines. Colors of individual data points indicate virus clade. **c** Neutralization breadth-potency plot summarizing the data in **b**. **d** Neutralization breadth of V3-crown bnDs and nAbs depending on their concentration according to data in **b**. **e** Dendrogram based on Spearman correlation (Supplementary Data 2) and single linkage hierarchical clustering depicting the similarity of neutralization fingerprints on the 40-virus panel for bnDs and a range of bnAbs and nAbs with different epitope specificity.

Starting from highly diverse DARPin libraries we performed five independent DARPin selections (Selections A-E) by Ribosome Display^32,33^. In these selections we utilized different combinations of Env-derived panning targets with the aim to select V3-specific DARPins with high neutralization breadth (Fig. 1a and Supplementary Figs. 1, 2 and 3a, b). From each ribosome display selection pool, individual DARPin clones were subsequently screened for binding to their respective selection targets and tested for cross-neutralizing activity (Fig. 1a and Supplementary Fig. 2). The best neutralizers of each selection were retained, yielding a final set of six DARPins (Supplementary Fig. 2b). V3 specificity was confirmed by the binding to linear V3 peptide (Fig. 1a). Sequence analysis showed that the selected DARPins were distinct clones that were neither related to each other, nor to the previously identified cross-neutralizing DARPin 5m3_D12^27^ (Supplementary Fig. 4a). All six selected DARPins were monomeric in solution (Supplementary Fig. 4b).

Subsequent analysis of neutralization breadth on multi-clade virus panels (Fig. 1, Supplementary Figs. 5a, 6 and Supplementary Data 1) identified three DARPins with exceptional breadth. They were accordingly named broadly neutralizing DARPins (bnDs). V3-crown specific DARPins 63_B7, SE4, and 5m3_D12 were less broad (Fig. 1a), but still surpassed the breadth of the prototypic V3-crown monoclonal antibody (mAb) 447-52D^34,35^. bnD.1, bnD.2 and bnD.3 reached 63%, 90 and 75% breadth, respectively, on a 40-virus multi-clade panel (Fig. 1b and c, Supplementary Data 1). Notably, these bnDs matched or even exceeded the breadth of the V3-base bnAbs PGT121 (70%), PGT128 (75%), and PGT135 (30%), however, without reaching their potency. V3-crown nAbs F425-B4e8, 3074 and DH753^19^ showed extended breadth when probed at concentrations equivalent to the bnDs, but were outperformed by bnD.2 and bnD.3 (Fig. 1c, d). Neutralization fingerprint analysis showed a strong positive correlation between the three bnDs, but not with V3-glycan bnAbs or V3-crown mAbs that, similar to bnDs, have lower potency (Fig. 1e and Supplementary Data 2). Modest significant correlations were observed between bnD.1 and the V3 mAb 3074, and between bnD.3 and 3074, the CD4bs mAb b12 and the interface bnAb PGT151 (Supplementary Data 2). Collectively, this suggests that the V3-crown specific bnDs form a novel class of broadly neutralizing agents.

### Broadly neutralizing V3-crown DARPins access V3 on Env post CD4 engagement

**Fig. 2:**
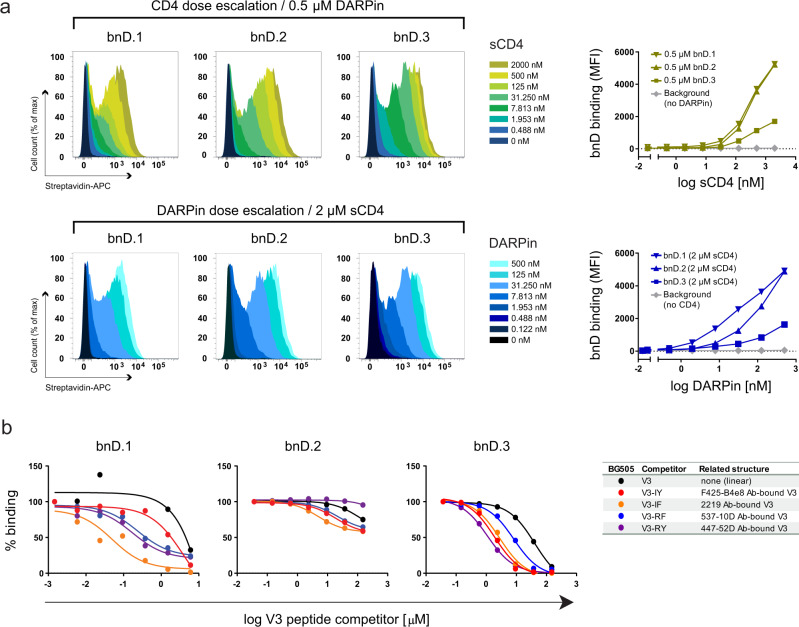
V3 DARPins depend on CD4 triggering to bind native HIV-1 Env. **a** Binding of biotinylated DARPins to cells expressing Env (JR-FL) was assessed by flow cytometry. Histograms of normalized fluorescence intensities and corresponding plots showing mean fluorescence intensities (MFI) as a function of concentration are depicted. Top: DARPin (0.5 µM) binding in the presence of increasing concentrations of sCD4. Bottom: DARPin binding at increasing concentrations in the presence of 2 µM sCD4. Background binding was assessed on cells expressing Mulv Env (shown are representative MFI data for the three bnDs). **b** Competition ELISA to probe for V3-crown conformational preferences of DARPins. DARPin binding to immobilized recombinant Env-derived proteins was measured at a concentration just before reaching saturation. Env proteins were chosen to maximize the dynamic binding range for the individual DARPin (bnD1: BG505gp120∆V1V2; bnD.2: JR-FLgp120; bnD.3: trimeric BG505-SOSIP). Each DARPin/Env pair was competed with increasing concentrations of the BG505 V3 peptides indicated in the legend.

Since the V3-crown is well shielded in the closed prefusion conformation of the native Env trimer, inhibitors that interact with the V3 must either be able to bypass V1V2 shielding or access the V3 during the entry process, when V1V2 is displaced upon CD4 engagement. We therefore probed the capacity of bnDs to bind native Env of the Tier-2 strain JR-FL expressed on 293-T cells, in the presence or absence of sCD4 (Fig. 2a and Supplementary Fig. 7). All V3-crown DARPins lacked the ability to bind native Env, unless triggered by sCD4. DARPin binding increased with higher concentrations of sCD4, suggesting that the virus-neutralizing capacity of the V3-crown bnDs lies in their ability to access V3 post CD4 triggering.

To rate the dependency on an open Env conformation, we compared the capacity of bnDs and prototypic V3-crown directed mAbs (F425-B4e8, 3074, DH753, 2219, 10A37, 1–79 and 447-52D) to neutralize wild-type (wt) and corresponding V1V2-deleted viruses (Supplementary Figs. 5b, 8, Supplementary Data 4). The gain in neutralization potency in the absence of V1V2 was dramatic for mAbs (ranging from 922 to 9,708 fold difference in geometric mean IC_50_ ratio (wt-Env/ΔV1V2-Env), but comparatively modest for bnDs (geometric mean IC_50_ difference of 30-, 15-, and 184-fold for bnD.1, bnD.2 and bnD.3, respectively).

Thus, despite their dependence on trimer opening, the V3-crown bnDs appear to have a considerable capacity to bypass V1V2 shielding. Opening of the trimer upon CD4 engagement occurs gradually with V1V2 retaining some shielding activity^36^. In line with this model, the V3 antibodies gained substantially more than bnDs from V1V2 deletion, suggesting that bnDs may already access the V3 at an earlier stage of the CD4 triggering when the trimer is not yet fully opened^36^.

This capacity of bnDs is likely favored by their small molecular size. Indeed, Fab fragments of the probed V3 crown mAbs gained neutralizing potency in the absence of V1V2 to a similar extent as bnDs (ranging from 68- to 459-fold, Supplementary Fig. 8a, b, Supplementary Data 4). Overall, Fabs showed higher potency against V1V2-deleted viruses compared to bnDs but lacked in activity against wild-type viruses, where bnD.2 and bnD.3 reached higher breadth (Supplementary Fig. 8c). Together this suggests that a particular capacity to bypass V1V2 shielding, rather than binding affinity, shapes the neutralization capacity of bnDs.

Due to their rigid interaction surface, DARPins often bind to a specific conformation of their target, as exemplified by the cross-neutralizing V3 DARPin 5m3_D12^26,27,31^. We thus examined the structural preferences of the V3-crown bnDs by comparing their reactivity with a linear V3-crown peptide and four structurally constrained V3-crown mimetic peptides. The V3-crown mimetic peptides (named V3-IY, V3-IF, V3-RY, V3-RF according to residues forming inter-strand hydrogen bonds) were designed to match distinct β-hairpin conformations of the V3-crown that had previously been identified in complexes with V3-crown mAbs^23,27,37–40^ (Supplementary Fig. 3a, b). Unlike DARPin 5m3_D12, all three bnDs reacted well with diverse V3 peptides immobilized to a solid phase, including V3 mimetics and linear V3 (based on sequences from the reference clade A strain BG505 and the clade B strain MN) (Supplementary Fig. 3c and Supplementary Data 3). Overall, bnD.3 displayed the strongest binding to the diverse V3-crown peptide variants. Competition binding in solution revealed, however, distinct conformational preferences (Fig. 2b and Supplementary Fig. 3d). For the bnDs, the strongest competition was generally observed with a structurally defined V3-crown mimetic, which outperformed linear V3-crown peptides in all comparisons. From these observations, we conclude that the structure of the V3 has a contributing role in bnD binding. For bnD.1 and bnD.2, the strongest competition was observed for mimetic V3-IF, and bnD.3 favored V3-RY, V3-IY, and V3-IF.

### Crystal structures of the V3-crown DARPin: epitope complexes reveal two distinct V3 conformations linked to broad neutralization

**Fig. 3:**
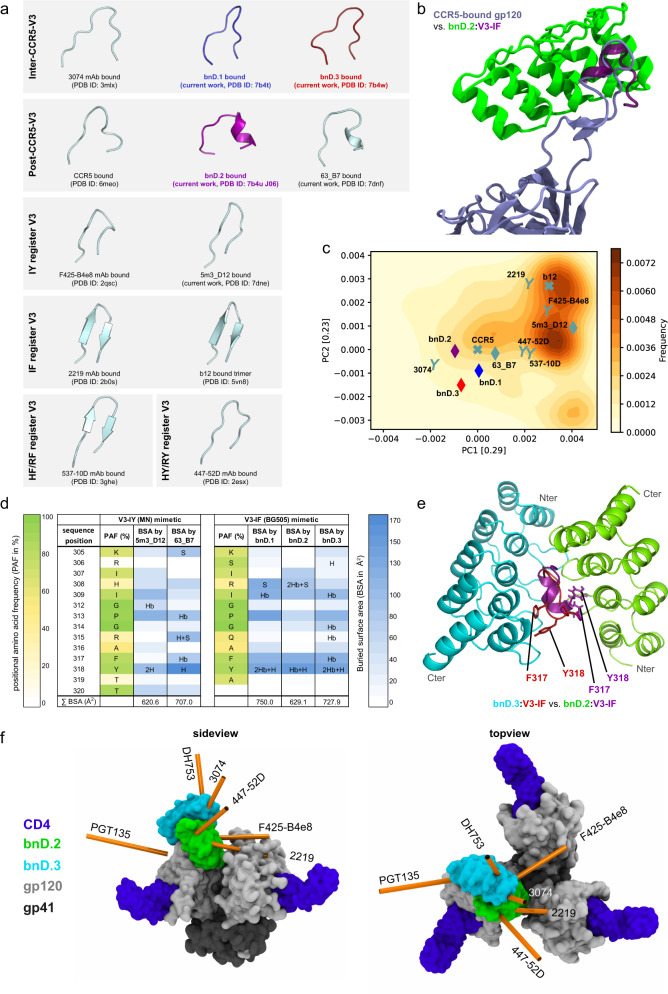
V3-crown conformations recognized by bnDs and binding modes. **a** Comparison of V3 conformations in complex with DARPins, mAbs, the CCR5 co-receptor, and in the partially open (b12-bound) Env trimer. 5m3_D12 and 63_B7 were co-crystallized with V3-IY (MN). bnD.1, bnD.2, and bnD.3 were co-crystallized with V3-IF (BG505). **b** Structural alignment of V3-IF (plum) in complex with bnD.2 (green) and CCR5-bound V3 (ice blue) (PDB ID: 6meo). For clarity, the D-Pro-L-Pro template of the V3-IF mimetic was omitted from the representation. **c** Principal component analysis for the conformational space sampled by the unliganded V3-crown during a molecular dynamics simulation of sCD4-bound, glycosylated gp120 initiated from the CD4 and CCR5-bound conformation (PDB ID: 6meo). Contour levels depict the frequency of adopted V3 conformations. The position of experimentally determined liganded V3 structures in the conformational space are marked by a diamond (V3-crown DARPin), Y (V3 mAb), or X (CCR5 co-receptor or CD4-binding site mAb b12) and labeled with the name of the respective ligand. Coloring according to **a**. **d** Summary of DARPin:V3-crown peptide interactions observed in the crystal structures listing the distribution of the buried surface area (BSA) on V3, total BSA, H-bonds (H), and salt-bridges (S). (Hb) indicates H-bonds with the V3 peptide backbone. The positional amino acid frequency (PAF) indicates how often each amino acid in the V3 peptide occurs in HIV-1 strains across group M (the V3 consensus sequence of group M is identical to V3 of strain BG505) (Supplementary Fig. 5). **e** Visualization of the interaction interface of bnD.2 (green) and bnD.3 (cyan) by structural alignment of the V3 peptides in PyMol. The side chains of key contact residues on V3 used by both bnDs are depicted with sticks. **f** Binding of V3-directed bnDs and mAbs modeled on the open Env trimer structure (bound by CD4 and the co-receptor-mimicking mAb 17b, PDB ID: 5vn3^11^. Surface representation of gp41 (dark grey), gp120 (light grey), CD4 (purple), bnD.2 (green) and bnD.3 (cyan). The approach angles of the mAbs were computed by principal component analysis based on the structural coordinates of each Fab-fragment and are indicated with orange rods.

In order to define V3-crown epitopes that lead to broad neutralization, we co-crystallized bnDs with V3-IF (BG505), i.e., the V3-crown mimetic that all bnDs bound best (Fig. 3, Supplementary Fig. 9 and Supplementary Data 5). 5m3_D12 and 63_B7 were co-crystallized with V3-IY (MN). Since 63_B7 also bound well to the linear peptide, it was additionally crystallized in complex with linear V3-crown peptide (MN). The canonical DARPin structure with the adjacent ankyrin repeats was maintained in all analyzed complexes (Supplementary Fig. 9a, c, e, g).

As expected from the pronounced preference of 5m3_D12 for V3-IY register mimetics (Supplementary Fig. 3c^27^), the structure of the isolated V3-IY mimetic was also retained in complex with 5m3_D12 (Fig. 3a and Supplementary Fig. 9a). In contrast, V3-IY and V3-IF mimetics in complex with the three bnDs and 63_B7 adopted conformations that were different from their original design (Supplementary Fig. 3a, b). In all cases, a loss of the mimetic’s β-hairpin conformation was observed (Fig. 3a). Two distinct V3-crown conformations emerged, each captured by two different DARPins (Fig. 3a, Supplementary Fig. 9e, g). The V3 conformation adopted in complex with bnD.1 and bnD.3 proved highly similar to the structure of linear V3 peptide bound by mAb 3074 (^5^, PDB ID: 3mlx, Fig. 3a and Supplementary Fig. 9g). In contrast, V3-IF in complex with 63_B7 and bnD.2 adopted a conformation strongly resembling the cryo-EM structure of the V3-loop on gp120 when bound to co-receptor CCR5 (^2^, PDB ID: 6meo) which features a helical turn in the C-terminal strand (Fig. 3a, b and Supplementary Fig. 9e). Considering this similarity, the conformation of V3 bound to bnD.2 will hereinafter be referred to as Post-CCR5-V3. Since bnD.1 and bnD.3 also target the V3 post-CD4 engagement, the conformation adopted by V3 in complex with these DARPins must either be a transitory intermediate state that occurs prior to CCR5 binding or an induced fit upon binding an intermediate. This conformation will hereinafter be referred to as Inter-CCR5-V3.

The V3-crown conformational space was investigated by performing a molecular dynamics (MD) simulation of fully glycosylated gp120 bound by CD4 (based on the CD4 and CCR5 bound gp120 PDB ID: 6meo^2^). A principal component analysis (PCA) of the conformations sampled by the V3-crown was then carried out. The V3-crown structures determined in complexes with DARPins or antibodies were projected into the eigenspace defined by the first two principal components (Fig. 3c). MD simulation suggested a high conformational flexibility of the V3 loop with β-hairpin variants being adopted most frequently and covering all experimentally determined V3 structures. Noteworthy, we observed that the Post-CCR5-V3 conformation is occasionally sampled spontaneously, possibly promoting the interaction with either CCR5 or bnD.2 (Fig. 3b). In contrast, the Inter-CCR5-V3 conformation was sampled less often in the MD simulations, suggesting that bnD.1 and bnD.3 must have the means to efficiently interact with V3 prior to the adoption of this V3 state.

The capacity of the bnDs to induce a fit or capture a transient conformation (conformational selection) was supported by additional observations. MD simulations showed that the conformational space of the V3-crown mimetics and the linear V3-crown peptide is strongly reduced when compared to the V3 loop on gp120 (Supplementary Fig. 10d–h). This is in agreement with Nuclear Magnetic Resonance (NMR) data of the V3-crown mimetics^27^ and confirms that mimetics only rarely adopt the Post-CCR5-V3 conformation. Despite this observation, the different V3-crown mimetics, as well as linear V3-crown peptide, were able to adopt the Post-CCR5-V3 conformation when in complex with 63_B7 and bnD.2 (Fig. 3b and Supplementary Fig. 9c, e). It should be noted that 63_B7 and bnD.2 were selected using different panning targets (V3-IY mimetic and CD4-bound gp120, respectively), thus highlighting again that a common transient conformation of the V3 must exist.

### bnDs engage conserved residues in the V3-crown

The analysis of the buried surface area (BSA) of the V3-crown mimetics across DARPin-complexes revealed several important features in the interaction motifs (Fig. 3d and Supplementary Data 6). Despite different preferences for the Inter- or Post-CCR5-V3 conformation, the bnDs overlapped in their contact patterns, engaging mainly with R/H308, I309, P313, F317, and Y318. All these residues are highly conserved across HIV-1 strains (>79%) except for position 308 where both arginine (42%) and histidine (31%) are frequently present (Supplementary Fig. 6a). The R308 side chain engages via a salt bridge with bnD.1 and bnD.2 (Fig. 3d, Supplementary Fig. 9f, h). Despite this contact, the bnDs maintained neutralization capacity against viruses with different residues in position 308 (Supplementary Data 1, Supplementary Figs. 5 and 6b), indicating the energetic importance of the other interactions. 63_B7 was the only DARPin that made substantial contact with the side chain of R315, which is prevalent only in clade B strains^41^. This would explain the restriction of 63_B7 for this clade (Supplementary Data 1). H-bonds formed with V3-crown peptide side chains and backbone differed substantially across DARPins (Fig. 3d and Supplementary Fig. 9). However, a striking commonality found across all DARPins was H-bonding with Y318.

Overall, we observed that bnD.3 forms eight H-bonds, distributed across six V3 amino acid residues, thus potentially leading to the comparatively high binding affinity observed for this DARPin (Supplementary Fig. 3c, and Supplementary Data 3 and 4). Six of these bonds were established with the V3-crown peptide backbone, and as a result, this should make bnD.3 less sensitive to V3 sequence variations. These features probably contribute collectively to the observed higher neutralization breadth of bnD.3 compared to bnD.1, which covers a BSA similar to bnD.3, but only forms four H-bonds with V3. In addition to the hydrogen bonds, additional hydrophobic contacts add to the interaction strength (Supplementary Fig. 9b, d, f, h, i).

### bnDs approach the V3-crown loop from opposite sides

The bnDs make extensive contacts with Y318 and F317 which are exposed to opposite sides of the V3 loop in the Inter- and Post-CCR5-V3 conformations, due to the presence or absence of the helical turn. This enables bnD.2 to approach the V3-crown from the opposite side compared to bnD.1 and bnD.3, and to interact with several shared conserved contact residues on the V3-crown (Fig. 3d and e).

In order to compare the relative orientations of the bnDs, Abs and Env, we carried out docking experiments incorporating the structural flexibility of the V3 loop sampled by the MD simulation of the glycosylated gp120 bound to CD4 (Fig. 3f and Supplementary Fig. 11). The bnD.3/bnD.1/3074 epitope is exposed to the outside of the trimer with mAb 3074 approaching the trimer from the top similar to DH753. In contrast, the epitopes of mAbs 2219 and F425-B4e8 face the two neighboring protomers. This allows only for a shallow approach angle of the Abs which, nevertheless, allows access for notable neutralization activity (Fig. 1c, d and Supplementary Fig. 8c). This provides further support for the accessibility of the bnD.2 epitope on functional Env, as bnD.2 binds the V3 from the same side as V3-crown Ab 2219 (and 447-52D)^5,19,25^.

### Functional mapping of the V3 DARPin epitopes

**Fig. 4:**
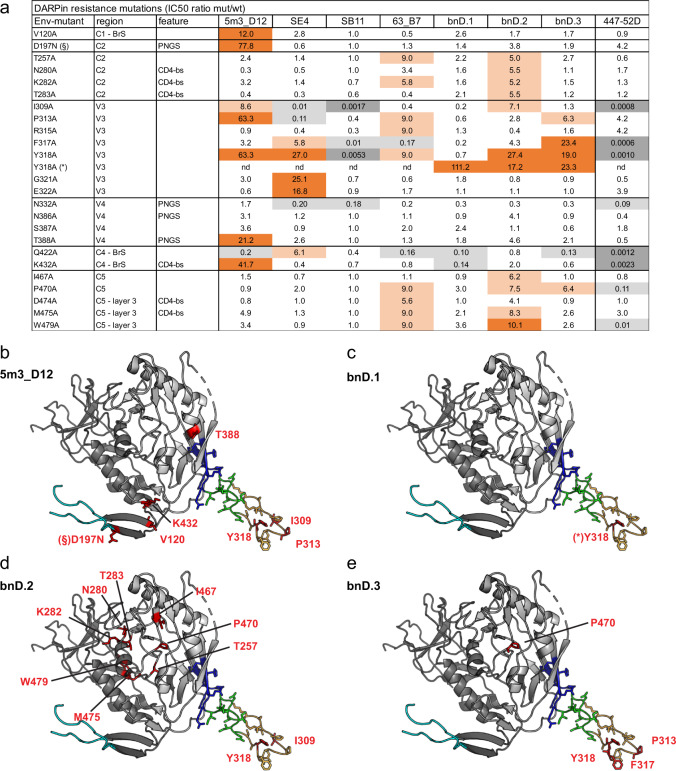
Mutational scanning reveals key residues that determine sensitivity to V3 bnDs. Mutational scanning of gp120 was performed across a 130 Env-pseudovirus mutant panel (Supplementary Data 7 and 8). **a** Overview of Env mutants that lead to >5-fold increase in IC_50_ compared to wt Env for at least one DARPin. For the DARPins, IC_50_ values for each mutant represent geometric means from at least two independent experiments (Supplementary Data 8). IC_50_ ratios (IC_50_mutant/IC_50_wt) are depicted. Light orange 5 < IC_50_-ratio<10; orange IC_50_-ratio>10; light grey 0.01 < IC_50_-ratio<0.2; dark grey IC_50_-ratio<0.01. PNGS: potential N-glycosylation site; CD4-bs: CD4-binding site; BrS: bridging sheet; C1-C5: Env constant regions 1-5; V1-V5: Env variable regions 1-5. **b-e** Structure of gp120 as in complex with sCD4 and CCR5 (PDB ID: 6meo; gp120 inner domain (dark grey), gp120 outer domain (light grey), V1V2 base (cyan), V3-base (dark blue), V3-stem (green), V3-crown (orange). Amino acid substitutions leading to a > 5-fold increase in the respective DARPin IC_50_ compared to the wt Env are indicated in red. (*/§): Mutations with >5-fold increase in IC_50_ identified in different Envs: BG505 (*) or JR-FL (§). JR-FL naturally lacks the glycosylation site at position 197.

To probe the functional relevance of individual amino acids for neutralization by the V3-crown DARPins, we conducted an Env mutational scanning analysis encompassing 130 point mutants within gp120 (Fig. 4, Supplementary Data 7). V3-crown mAb 447-52D was included for comparison. A range of Env single-residue mutants showed enhanced neutralization sensitivity to V3-crown directed agents, indicating a more open Env trimer structure with increased V3 exposure (e.g., I309A, F317A, Y318A, Q422A, and K432A, see Fig. 4a and Supplementary Data 7). Notably, mAb 447-52D benefited from improved access to a much higher extent than the bnDs, reminiscent of the effect of V1V2 deletion (Supplementary Fig. 8b) suggesting that antibody access may have a greater dependence on complete trimer opening than bnDs.

Resistance mutations, defined by a > 5-fold increase in IC_50_ of mutant over wild-type virus, were mapped onto the crystal-structure of gp120 complexed with CD4 and CCR5^2^ (Fig. 4b–e). Despite shared contact residues (Fig. 3d), identified resistance mutations showed comparatively little overlap across DARPins. Only Y318 proved to be critical for most DARPins, but also for this residue influence varied depending on the Env context (Fig. 4a).

Five shared resistance mutations highlighted the similarity between bnD.2 and 63_B7 (Fig. 4a and Supplementary Data 7). These positions (T257, K282, P470, M475, W479) localize outside V3 near the interface of the gp120 inner and outer domains and involve residues of the CD4 binding site and/or layer 3, a structural element previously implicated in Env conformational transitions^42^. Mutation of the neighboring residues N280 and D474 also showed an effect on bnD.2 and 63_B7, although the 5-fold threshold was not reached in both cases. The P470A mutation also influenced bnD.3 potency but stayed below the 5-fold threshold for bnD.1.

The removal of glycosylation sites surrounding the V3 loop (N197, N332, N386) had hardly any effect on bnD activity (Fig. 4a and Supplementary Data 7). Observed decreases in sensitivity were less than 5-fold. Removal of N332 led to higher sensitivity to all three bnDs but the increase was less than 5-fold. We only noted a potential effect of glycosylation on 5m3_D12: Introduction of a glycan at N197 strongly decreased sensitivity, conversely the T388A substitution (and to a lower extent also N386A) led to a decrease in sensitivity, suggesting that loss of this glycan may lead to altered structural dynamics of the V3 loop that do not favor 5m3_D12 binding. In support of this, the V3-IY conformation preferred by 5m3_D12 was only rarely sampled in a V3 MD simulation on deglycosylated gp120 (Supplementary Fig. 10).

### Accessibility of V3-crown bnD epitopes

**Fig. 5:**
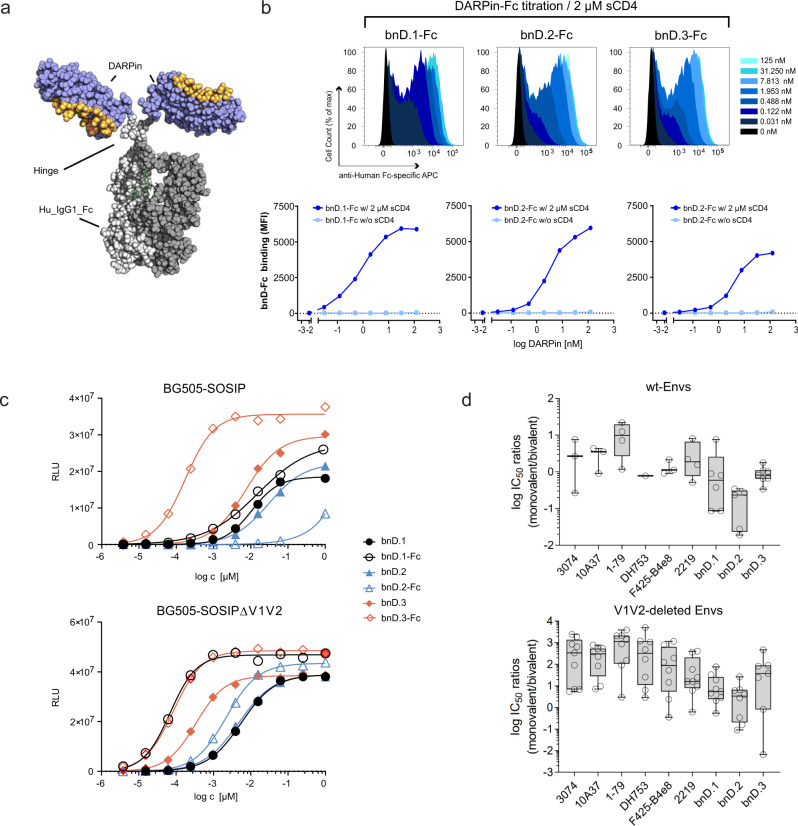
Activity of bnDs as bivalent molecules. **a** Model of a bivalent DARPin-Fc generated by fusing the DARPin (violet with orange interaction surface) to the N-terminus of the Fc-region of human IgG1 (light grey and dark grey for the paired chains) with a preserved hinge region. **b** Binding of bivalent DARPin-Fc fusions to cell surface-expressed Env (JR-FL) measured by flow cytometry. DARPins-Fc fusions were titrated and incubated with cells in the presence or absence of 2 µM sCD4. Mulv Env served as a negative control. Histograms of normalized fluorescence intensities (top panel) and dose-response curves with mean fluorescence intensities (MFI, bottom panel) are depicted. See also linked experiments in Supplementary Fig. 12. **c** Binding of increasing concentrations of monovalent DARPins and bivalent DARPin-Fc fusions to wt (BG505-SOSIP) and V1V2 deleted Env trimer (BG505-SOSIP∆V1V2) in ELISA. Representative relative luminescent unit (RLU) data from one of two independent experiments are shown. **d** Comparison of monovalent and bivalent V3 inhibitors. Log IC_50_ ratios (Fabs/mAbs and bnDs/Fc-bnDs) from a panel of 9 Tier-2 viruses (top) and corresponding V1V2-deleted viruses (bottom) are depicted. Values > 1 indicate higher potency of the bivalent inhibitor version. Individual data points are shown as circles and were calculated from geometric means of two independent experiments for wt and V1V2-deleted virus respectively (*n* = 2). Pairs with resistant strains were not included in the plots. Box plot limits extend from the 25th to 75th percentiles; centerline: median; whiskers indicate the minimum and maximum values. See Supplementary Data 9 and Supplementary Fig. 13 for a full data set.

Due to their smaller size, DARPins (~16 to 20 kDa, depending on the number of ankyrin repeats, see Supplementary Fig. 4) may access restricted sites more easily than Abs (~150 kDa). In order to assess the influence of space constraints, we generated bivalent Fc fusion proteins of each bnD (Fig. 5a; bnD.2-Fc: ~80 kD; bnD.1-Fc and bnD.3-Fc: ~85 kD). Like the monovalent DARPins, bnD-Fcs required CD4 triggering to bind cell surface-expressed JR-FL Env (Fig. 5b) while V3-crown mAbs display a low-level binding also in the absence of CD4 at equivalent concentrations (Supplementary Fig. 12). We next compared bnD-Fc binding to a recombinant native-like Env trimer, BG505-SOSIP^8,43,44^, which is known to partially expose V3^44^ (Fig. 5c, Supplementary Data 9). The effect of bivalency on binding V3 on BG505-SOSIP varied considerably across bnDs. While the binding for the bnD.3-Fc improved substantially, bnD.1-Fc binding activity remained unchanged and bnD.2-Fc even recorded a strong loss in binding capacity. Binding to trimeric BG505-SOSIP lacking V1V2 improved for bnD.1-Fc and to a lesser extent for bnD.2-Fc over the monovalent DARPin version, while bnD.3-Fc gained less in activity compared to binding to the wt protein (Fig. 5c, Supplementary Data 9). Collectively, this suggests that bnD.1 epitopes are arranged such that a bivalent engagement across the trimer is not possible, and that the bnD.2 epitopes are not accessible for the larger sized bnD.2-Fc in its current design. Neutralization efficiency of the bnD-Fcs against wt and V1V2-deleted viruses revealed a similar pattern (Fig. 5d, Supplementary Fig. 13, and Supplementary Data 9). Unlike bnD.2-Fc, bnD.1-Fc showed a moderate, bnD.3-Fc a stronger consistent increase in potency compared to their monovalent counterparts against V1V2-deleted viruses and the neutralization sensitive (Tier-1) strain SF162. Remarkably, when probing neutralization of difficult-to-neutralize (Tier-2) wt viruses, bnD.3-Fc fully retained its activity while bnD.1-Fc and bnD.2-Fc showed less activity than their monovalent counterparts. Interestingly, bivalent V3-crown mAbs had a modestly higher potency against wild-type Env compared to their monovalent Fab version while bivalent binding strongly improved potency against V1V2-deleted Env (Fig. 5d). Thus, although wild-type Env not only restricts access to the V3 but also bivalent binding, full-sized V3-crown Abs performed on average better than corresponding Fab molecules.

Collectively this indicates that both for V3-crown Abs and bnDs the exposure of the epitope during the entry process rather than the size of the inhibitor regulates neutralization efficacy. The analysis of bivalent bnD-Fc binding and neutralization activity highlights the overlapping influences of avidity gain and steric constraints. The observed differences amongst the three bnD-Fcs are in agreement with our docking studies which placed the bnD.1/bnD.3 epitopes to the outside of the trimer and thus more accessible than the bnD.2 epitope located between two protomers. Further space constraints for bnD.2-Fc may result from the C-terminal linked Fc facing the host-cell membrane (Fig. 3e and f). These constraints may in part be specific to the design of the bnD.2-Fc construct, as the bnD.2 epitope is accessible for larger sized inhibitors such as mAb 10A37 (Fig. 5d and Supplementary Fig. 8c) which recognizes a similar V3 conformation than bnD.2^45,46^, and mAb 2219 which approaches V3 from a similar angle (Fig. 3f). Of particular note, even as an 85-kD Fc fusion protein, bnD.3 showed sustained neutralizing potency, underlining that the V3-crown can be effectively targeted by larger agents.

### Distinct patterns of V3-crown plasma antibody reactivity are linked with neutralization breadth

We next investigated whether naturally occurring V3-crown Abs exist that share with bnDs a preference for distinct V3-crown conformations. Probing a range of V3-crown mAbs we found that most, but not all, bind better to the linear V3-crown peptide than the four V3-crown mimetics (Supplementary Fig. 14a). Reactivity of the mAbs towards the mimetics differed, with most mAbs binding the V3-crown mimetics V3-IY and/or V3-IF better than V3-RF and V3-RY, further illustrating conformational preferences. Only mAb 2442 bound all mimetics with similar affinity.

**Fig. 6:**
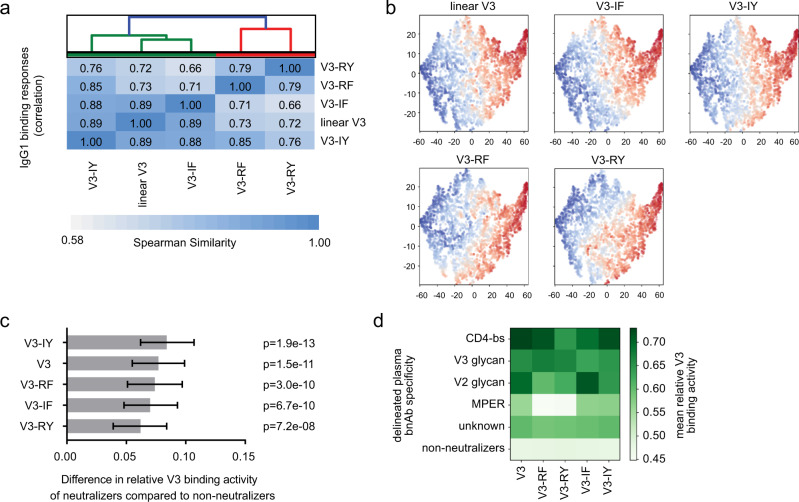
The V3-crown IgG1 response in HIV-1 infection recognizes distinct V3-crown conformations and is linked with the development of neutralization breadth. Relative plasma IgG1 binding activity (distributed uniformly in [0,1]) to linear V3 and structurally constrained BG505 V3-crown mimetics (V3-IY, V3-IF, V3-RF, V3-RY) from 4281 chronic HIV-1 infected individuals enrolled in the Swiss 4.5 K Screen^13, 47^ were obtained and assessed in the context of neutralization activity data of the cohort (defined as a cross, broad and elite neutralization, or no neutralization) available from^47^. Source data are provided as Supplementary Data 10. **a** Unweighted average linkage hierarchical clustering based on the Spearman correlation of relative V3-crown peptide IgG1 binding activities across the cohort indicates reactivity clusters dependent on V3-crown peptide conformation. **b** A two-dimensional representation (t-SNE map) of all 4,281 plasma samples based on relative IgG1 binding activities to the five V3 peptides. Red color denotes high binding activity and blue color denotes low binding activity to the V3 peptide indicated on the top of each panel. **c** Comparison of relative V3 binding activity in plasma from patients with (*n* = 909) and without (*n* = 2250) heterologous virus-neutralizing activity by multivariable linear regression analysis. Grey bars indicate the mean difference in relative binding activity for individual peptides (two-tailed p-values from t-test are provided; they are not adjusted for multiple testing). Black error bars indicate the 95% confidence intervals. These data belong to a comprehensive analysis of host, virus, and disease parameters, shown in Supplementary Fig. 14c and Supplementary Data 11, where only the *n* = 3159 patients with complete information on all parameters were included. **d** The top 105 neutralizing plasmas of the cohort were stratified by bnAb epitope specificity (using neutralization fingerprinting^47^) and their mean relative V3 binding activities were compared.

We next leveraged the well-characterized patient cohort of the Swiss 4.5 K Screen^13,47^ to assess a potential impact of V3 reactivity on neutralization breadth. This cohort comprises 4281 individuals with chronic HIV-1 infection for whom detailed information on plasma neutralization activity, anti-HIV binding Abs, patient demographics, and disease parameters have been established^13,47^. First, we compared the binding reactivity of plasma IgG1 to linear V3-crown peptide (strain BG505) and the four corresponding V3 mimetics (V3-IY, V3-IF, V3-RY, and V3-RF) across the cohort (Fig. 6 and Supplementary Fig. 14b). Correlation analysis showed that the relative V3-crown binding activities formed two reactivity clusters, one comprising the linear V3-crown peptide together with V3-IY and V3-IF and the other comprising V3-RY and V3-RF (Fig. 6a), underscoring that V3-crown antibodies with different conformational binding preferences occur in HIV-1 infection.

Two-dimensional V3 response maps based on the dimensionality reduction algorithm t-SNE showed distinct areas with high V3 reactivity that overlapped between V3 probes (Fig. 6b). Visualization of V3 patterns in sub-groups based on neutralization activity revealed no distinct clustering (Supplementary Fig. 15). Cross, broad, or elite plasma neutralization capacity and top 105 neutralizers stratified according to their predicted bnAb specificity as defined in^47^ showed overall no strong accumulation in V3 dense areas of the t-SNE plots. A degree of clustering was however evident amongst certain bnAb specificities amongst top neutralizers, in particular for trimer-apex predicted plasmas (Supplementary Fig. 15b). As we previously observed that trimer-apex (V2) responses are more prevalent outside subtype B^47^ and these plasma should react better with the subtype A (BG505) based antigens in binding, this accumulation in the t-SNE plots has to be expected and alone cannot define an association between bnAb activity and V3 binding responses.

To control for influences of co-variables we next conducted a multivariable linear regression analysis analyzing the influence of patient and disease parameters on relative binding activities to V3 probes (linear V3 and mimetics) and Env targets (gp120 and trimer) (Supplementary Fig. 14c). Previous analyses of the Swiss 4.5 K Screen identified an influence of infection length, viral load, virus diversity, and black ethnicity on the development of neutralization breadth and IgG responses to HIV-1 Env antigens^13,47^. Here, we found that length of untreated infection and transmission by injection drug use (which entails prolonged periods of untreated HIV-1 infection), correlate positively with IgG1 V3 binding activity across all tested V3 probes (Supplementary Fig. 14c). Viral load, which correlated strongly with trimer and gp120 IgG1 responses was not associated with increased V3 antibody responses, highlighting epitope-specific differences in the parameters that steer binding antibody responses, as also noted by Kadelka et al.^13^. As noted before, the reactivity of plasma with the Env antigens proved to be subtype-dependent, with subtype B plasmas showing higher reactivity with subtype B derived antigens (MN and JR-FL) and non-subtype B plasmas showing higher reactivity with BG505 antigens. Likewise, we confirmed black ethnicity as a driver of IgG1 binding responses including V3 responses (Supplementary Fig. 14c and Kadelka et al.^13^). Most intriguingly, when comparing Swiss 4.5 K patients with and without neutralizing activity by multivariable linear regression, we found that neutralizing plasma activity was associated with higher IgG1 binding activity for V3-crown peptides, with the strongest association observed for reactivity with the V3-IY mimetic (Fig. 6c).

This higher prevalence of V3-crown-specific antibody responses among broad neutralizers is intriguing and suggests that V3 responses are influenced by the same disease parameters as bnAbs, but may evolve independently and can serve as surrogate markers for the evolution of bnAbs. Alternatively, V3-crown responses may be functionally linked to the evolution of bnAbs. For example, escape of V3-crown Abs may drive the evolution of different bnAb types. In addition, rare V3-crown mAbs may themselves exhibit notable neutralizing breadth.

To gain further insight into the potential functionality of V3-crown responses, we next examined whether V3 reactivity was uniformly high among bnAb inducers or whether differences existed depending on the bnAb specificity elicited again utilizing the top neutralizers with predicted bnAb specificity (Fig. 6d). Intriguingly, we found that V3 reactivity differed depending on the bnAb specificity of the plasma with CD4bs bnAb predicted plasma recording the highest V3 reactivity. V3-glycan bnAb reactivity was associated likewise with high V3 peptide reactivity across all probes, whereas V2-glycan bnAb plasmas showed high, but differential reactivity across V3 probes. Plasmas with yet unidentified bnAb specificity and MPER bnAb predicted plasmas showed lower V3 reactivity than the other bnAb plasmas but also for these plasmas the reactivity was higher than in non-neutralizers in agreement with the analysis in Fig. 6c.

In conclusion, the population-wide survey of V3-crown responses in the Swiss 4.5 K cohort (*n* = 4281) lends support to the idea that the naturally occurring antibody response in HIV-1 infection is shaped to distinguish conformational differences of V3 (Fig. 6a). The V3-crown response appears to co-evolve with neutralization breadth (Fig. 6c and d), highlighting the importance of deciphering which V3 responses are an irrelevant by-product and which are a direct or indirect component of bnAb evolution.

## Discussion

Harnessing the potential of the V3-crown for HIV-1 inhibition has been attempted for several decades. Termed initially the principal neutralization domain, it rapidly became evident that V3-crown neutralization by antibodies is rendered inefficient due to its conformational masking^9,48^. Moreover, the immunodominance of the V3-crown is thought to distract the antibody response from known bnAb epitopes by competition for T follicular helper cells during affinity maturation in germinal centers^49^. For these reasons current Env-immunogen design seeks to avert the immunodominant V3-crown response as exemplified by the new generations of well-shielded, stabilized, soluble Env trimer immunogens^50–54^. Nonetheless, exploiting the V3-crown remains a tempting target because of its functional importance, sequence conservation, and high immunogenicity — potentially all key factors for efficient inhibition. V3-crown antibodies show exceptional potency and cross-reactivity in the absence of V1V2-shielding^9^. Thus, the potential for effective inhibition is present, provided shielding can be bypassed.

In this study, we demonstrate that broad neutralization, reaching up to 90% of breadth, via the V3-crown can indeed be achieved by creating novel DARPin-based inhibitors. The large breadth of the V3-crown bnDs is even more astonishing as they are currently still substantially less potent than bnAbs targeting prefusion Env. The discovery of the bnDs provides proof that the V3-crown, although only transiently exposed on the open Env during viral entry, is widely targetable across divergent strains. The bnDs further define two conformations, Inter-CCR5-V3 and Post-CCR5-V3 that expose conserved residues and are accessible on the CD4-triggered Env trimer.

MD simulations suggest that the V3-crown samples various conformations before CCR5 engagement. Molecular docking suggests that the Inter-CCR5-V3 conformation, defined by bnD.1 and bnD.3, may thereby be exposed already on a partially open trimeric Env^11^ (Supplementary Fig. 11b). bnD.2 cannot access V3 on this Env conformation due to steric hindrance by V1V2. This bnD requires CD4-triggered Env with a more exposed V3 to bind. The capacity of bnD.2 to bind Env similarly to CCR5 in the Post-CCR5-V3 conformation may, however, be a key component of its extraordinary neutralization breadth. All three bnDs benefit less from V1V2 deletion than nAbs, supporting the notion that bnDs can access a comparatively early - partially - opened state of the trimer^36^. This notion is further strengthened by the fact that bnD potency does not benefit from point mutations opening the trimer to the same extent compared to V3 mAb 447-52D, for which we noted strongly enhanced neutralization potency. While V3-crown mAbs in part also access partially open Env, space constraints appear to restrict bivalent binding unless V1V2 shielding is completely released. Overall, this indicates that access is less of a limiting factor for neutralization by V3-binding bnDs than V3 antibodies, with the latter likely requiring complete and prolonged opening of Env to be fully effective.

The majority of bnAbs function by neutralizing free virus^36,55^. A notable exception is a group of bnAbs targeting the membrane-proximal-external-region (MPER) on gp41 that are known to require CD4 triggering to efficiently bind Env^56,57^. MPER bnAbs, like V3-crown bnDs, are remarkably broad, but not very potent^58^. Other bnAbs also exhibit post-CD4 activity, as demonstrated during cell-to-cell transmission, where likewise a broad but not very potent activity is seen^59,60^. The overall lower potency of post-CD4 neutralizing activity is with great certainty a consequence of the transient and brief exposure of these Env states. This makes the activity of V3 bnDs all the more remarkable. Intriguingly, bnAb VRC01 which failed to protect against HIV acquisition in the AMP trial^61^ has high potency against the free virus but not post attachment and during cell-cell transmission^59,60^. Combining bnAbs with high potency against the free virus with inhibitors that provide inhibition after binding to CD4, such as the novel V3-crown bnDs described here, would increase the options for effective entry inhibition and is worth exploring in forthcoming studies. Therapeutic development of DARPins has been demonstrated in other disease models^26,62–65^. DARPins are being evaluated in late-stage clinical trials for, e.g., COVID (ensovibep^62,66^), cancer (MP0310^67^), or neovascular age-related macular degeneration (abicipar^68^). DARPins can be refined by a large variety of engineering strategies increasing valency, potency, and half-life^26,69–79^,(and Supplementary Fig. 1).

CD4 triggering occurs gradually as demonstrated by a step-wise exposure of neutralization sensitive epitopes^36^. Access of the V3-crown bnDs to early opening Env intermediates, as our results suggest, maybe decisive for their activity, as it elongates their window of opportunity. Certain post-receptor engagement conformations may also be sampled by Env spontaneously and might be trapped by the bnDs^19,80^. We observed no exposure of the bnD epitopes in the absence of CD4 in our experimental setup using a relatively neutralization-resistant Env (Tier-2 strain JR -FL). However, spontaneous conformational sampling may differ among strains and needs to be taken into consideration^19,36,81^. Collectively, these observations demonstrate that post-CD4 conformations of Env may be more widely accessible for neutralization before and during the entry process than was previously thought and could be exploited. The description of inhibitor-targetable V3-crown conformations defined here is, therefore, an important step in this direction.

Although bnDs benefit from their smaller size in accessing the distinct V3 conformations post-CD4 engagement, the analysis of bivalent Fc-fusion constructs together with mAb-Fab-pairs highlights that larger molecules can also access these regions. Spatial constraints inflicted by the V1V2, host cell membrane, and neighboring Env protomers however differentially affect access to epitopes depending on the size and epitope of the inhibitors. As demonstrated by bnD.3, high affinity and avidity effects can counterbalance the access limitations of larger molecules. Therefore, antibodies could also benefit from these effects to attack this domain efficiently.

In this study, we linked strong V3-crown responses with neutralization breadth in chronic HIV-1 infection (Fig. 6b, c). Whether these binding reactivities are a surrogate response that evolves alongside bnAbs without having a direct effect, whether they are indicative of the evolution of rare V3-crown bnAbs, or whether they resemble a contribution of V3-crown Abs as helper lineages for other bnAbs, remains to be defined. A proof of principle that the V3 conformations recognized by the bnDs are also targetable by antibodies comes from structure analysis of two V3-crown mAbs that neutralize some Tier-2 viruses but lack overall breadth. MAb 10A37 targets a conformation closely related to Post-CCR5-V3 bound by bnD.2^45,46^. MAb 3074 recognizes a conformation of V3 similar to Inter-CCR5-V3 recognized by bnD.1 and bnD.3 and approaches V3 from the same side as these DARPins^5,19,20,82^. mAb 3074-like Abs may therefore benefit in particular from better accessibility of their epitope, similar to bnD.3/bnD.3-Fc.

Collectively, our findings strongly suggest that directing antibody responses to distinct V3-crown conformations accessible after CD4 attachment should be feasible, thus opening roads for developing vaccine strategies to elicit bnD-like V3-crown bnAbs. Exploiting this knowledge will likely require the design of appropriate epitope scaffolds to be used as immunogens in order to channel the polyclonal V3-crown response to the desired specificity. These responses will likely not be potent, but due to their high breadth and capacity to recognize an entry state in which bnAbs targeting prefusion Env have less activity, may add important functionality to multivalent vaccines.

## Methods

### Cells

HEK 293-T cells were obtained from the American Type Culture Collection and TZM-bl cells through the NIH AIDS Reagent Program. Both cell lines were cultured in DMEM containing 10% FCS. HEK 293 T Freestyle^TM^ suspension (293 F and Expi293F) cells (Thermo Fisher) for protein expression were maintained in suspension in serum-free FreeStyle^TM^ 293F and Expi293F Expression Media (both from Thermo Fisher) according to the manufacturer’s instructions. Media were supplemented with Penicillin and Streptomycin and cell lines were regularly checked for the absence of mycoplasma. No specific cell line authentication was performed.

### Reagents

Properties of monoclonal antibodies used in this study are listed in Supplementary Supplementary Data 12. We thank the individuals and agencies listed there as providers of antibodies and/or Ab expression plasmids for this study either directly or via the NIH AIDS Research and Reference Reagent Program (NIH ARP). Soluble four-domain CD4 (sCD4)^83,84^ was provided by W. Olson (Progenics Pharmaceuticals Inc., Tarrytown, New York, USA).

### Peptides and mimetics

CD4M47 was synthesized as described^85^. Linear V3-crown peptides and structure-constrained V3-crown mimetic peptides of strains MN, JR-FL, and BG505.W6M.ENV.C2 were synthesized as described^39^. The V3 mimetic peptides (Supplementary Fig. 3a, b) were designed to build anti-parallel β-strands that differ in the formation of inter-strand hydrogen bonds (mimetic registers). Four structural V3 loop mimetics with different registers were cyclized by a D-Pro-L-Pro (^D^PP) dipeptide which stabilizes the hairpin conformation. The codes of the registers (IY, IF, RY/HY, RF/HF) refer to the amino acids forming the respective hydrogen bond^27,39^. The IY register was defined in complex with mAb F425-B4e8^37^, IF in complex with mAb 2219^40^, RF/HF in complex with mAb 537-10D^38^, and RY/HY with mAb 447-52D^23^. See Supplementary Fig. 3a, b for a full overview of peptides and mimetics used.

Peptides were biotinylated for use in ELISA, Luminex-based assays, and Ribosome Display. The linear V3 (MN) peptide was biotinylated on the N-terminus of a four-glycine N-terminal spacer (Supplementary Fig. 3a). For V3-IY (MN), a PEG08 linker was introduced between the L-proline in the D-Pro-L-Pro template of the mimetic and biotin. In the same way, a PEG-4 linker was introduced between the peptide chain and biotin in all V3 mimetics based on the sequence of strain BG505 (see also^39^). In the case of linear V3 (BG505), a biotinylated PEG-4 linker was attached to an additional N-terminal glutamic acid residue. All synthetic peptides were ≥95% pure by analytical HPLC and displayed electrospray MS spectra consistent with the expected masses.

### Protein expression, purification and modification

#### CD4

Two-domain sCD4_183 protein was expressed in *E. coli* and purified as described in^36^.

#### Env proteins

Codon-optimized sequences of strain JR-FL gp120 wild-type and V1V2 loop deleted JR-FL^86,87^ were custom synthesized (GeneArt, Germany). The BG505-SOSIP source plasmid was kindly provided by J.P. Moore (Weill Cornell University, New York, USA) and Rogier Sanders (Academic Medical Center, Amsterdam, Netherlands)^44^. All Env constructs were fused to a C-terminal AviTag and cloned into a CMV/R expression vector^88^ to allow in vitro biotinylation. Env proteins were produced by transient transfection of HEK 293 T Freestyle suspension (FS) cells. BG505-SOSIP was expressed by transient transfection using a furin-expressing helper plasmid at a 3:1 ratio^89^. All HIV-1 Env proteins were purified from culture supernatants using *Galanthus nivalis* lectin resin (Vector Laboratories) as described in^90^. Mono-biotinylation with BirA enzyme was performed according to the manufacturer (Avidity, Aurora, USA). Proteins were subjected to Superdex 200 size exclusion chromatography (GE Healthcare, USA) to derive pure monomer or trimer.

#### Monoclonal antibody and Fab production

DNA-strings encoding the Fab regions of Abs 3074, DH753, F425-B4e8, 2219, 10A37 were synthesized (Geneart, Thermo Fisher Scientific) and cloned into human IgG1, human Igkappa, and human Iglambda expression vectors (AbVec) using In-Fusion methodology (Takara). Antibodies were expressed in Expi 293 F cells (Thermo Fisher Scientific) by transient transfection using TransIT-PRO transfection reagent (Mirus Bio LLC) according to the manufacturer’s instructions. Supernatants were harvested six days after transfection, sterile filtered, and supplemented with Protease Inhibitor tablets (Roche). Antibodies were purified from supernatants using AmMag Protein A Magnetic Beads (Genscript) according to the manufacturer’s protocol. After elution of the Abs by 0.1 M glycine, pH 2.7, the eluate was neutralized with 1 M Tris, pH 8.7 using a 20^th^ of the eluate volume. Abs were further purified on a HiLoad 16/600 Superdex 200 pg size exclusion chromatography column (GE Healthcare) equilibrated in 20 mM NaHPO_4_/NaH_2_PO_4_, 100 mM NaCl, pH 6.0, and concentrated to 6 mg/ml (40 µM) using Amicon centrifugal filter units (Millipore).

Fab fragments were prepared from purified mAbs by digestion with papain-agarose resin (Thermo Fisher Scientific) overnight at 37 °C using 50 µl settled resin/mg IgG in 20 mM NaHPO_4_/NaH_2_PO_4_, 10 mM EDTA, 20 mM cysteine, pH 7.0. To remove Fc-fragments and non-digested IgG the reaction was subsequently incubated with AmMag Protein A magnetic beads (Genscript) overnight at 4 °C, aiming for a five-fold excess in IgG binding capacity of the beads over the total input of IgG in the digest. The Fab fragments remaining in the supernatant were then further purified on a size exclusion chromatrography column (either HiLoad 16/600 Superdex 200 pg or Superdex 200 10/300 GL Increase (both from GE Healthcare)) equilibrated in either 20 mM NaHPO_4_/NaH_2_PO_4_, 100 mM NaCl, pH 6.0 or PBS, pH 7.4 and concentrated to >16 µM concentration using Amicon centrifugal filter units (Millipore).

Concentrations of mAbs and Fabs were estimated by measuring OD_280_ and applying specific absorption coefficients determined by ProtParam tool (www.expasy.org) using the respective amino-acid sequences. When converting concentrations, the molecular weight of the mAbs and Fabs was approximated to be 150 kDa and 50 kDa, respectively.

### DARPin selection by ribosome display

General description of the methodology: The principal methods of ribosome display, DARPin library design, and DARPin selection by ribosome display have been previously described in detail^26,28,31,33,91–93^. An overview of the methods is provided in Supplementary Fig. 1.

Ribosome display is an in vitro translation method, where genetic information (i.e., mRNA) and translated protein remain linked in a ternary complex to the ribosome^33^. Since the DARPin sequence is fused to a C-terminal additional sequence (tether), it emerges from the ribosome and folds, while the C-terminus is still linked as the peptidyl-tRNA and thus trapped in the ribosome. From the DARPin library, DARPins of interest are enriched, based on their binding properties, and simultaneously their coding sequence is obtained from the mRNA^33,91,92^. DARPin library screening in combination with ribosome display has been successfully used in diverse settings^26,32,91^.

DARPin 5m3_D12 was selected in a previous study^27^. The selection of novel V3-crown specific DARPins in the present study was performed by Ribosome Display (RD) as described previously^27,31,32,91,92^. In the five RD selections performed (selections A-E, Fig. 1a and Supplementary Fig. 2), the following proteins were used as panning targets: (i) the V3-crown mimetic peptide V3-IY(MN)^27^, (ii) monomeric gp120 arrested in the CD4-bound state, on which V3 is highly accessible (JR-FL-gp120 (clade B) in complex with the CD4 mimetic CD4M47^85^ and ZM651-gp120 (clade C) in complex with soluble CD4 (sCD4)), and (iii) BG505-SOSIP (clade A), an engineered trimeric Env gp140 ectodomain on which access to V3 is restricted by the native-like structural environment^8,43,44^. For each of the three target types, five to six RD selection rounds were conducted, including an off-rate selection round^32,70,91^, to enrich for high-affinity binders (Supplementary Fig. 2). In successive RD rounds either a single panning target was used or two different targets were employed in alternating fashion to promote the selection of cross-reactive DARPins (Supplementary Fig. 2).

Three versions of starting DARPin libraries were employed, each encoding N3C DARPins with one N-capping, one C-capping and three internal ankyrin repeats (Supplementary Figs. 1, 2, and 4a). DARPin selections A and B were conducted using a first-generation N3C library that encodes N3C DARPins with one N-capping, one C-capping, and three internal ankyrin repeats^29^. Selection C used an improved second-generation N3C library, while selections D and E used a combination of this latter library with an N3C-loop DARPin library^94^. In the second-generation N3C library and N3C-loop DARPin library, additional positions in both caps were randomized (Supplementary Fig. 4a)^26,94^, and a more stable C-cap was used^95^. The N3C-loop library furthermore contains, in addition to the randomized positions in the DARPin structure^29^, the insertion of a flexible loop with randomized residues^94^, potentially allowing additional types of target interaction, similarly to the complementary determining region 3 of Ab heavy chains^96^. The second-generation N3C library and N3C-loop DARPin library was also used in a version without randomized caps.

Manual Ribosome Display selection: Selection A and B were performed as described in^27^. Briefly, the respective biotinylated target (Supplementary Fig. 3a and b) was bound to streptavidin-coupled magnetic microbeads (MyOne-T1 Dynabeads, Invitrogen) and panning of ternary DARPin-mRNA-ribosome complexes was performed in 1.5 ml microtubes. Heparin, which is commonly used in RD to prevent non-specific mRNA binding, was omitted from the panning buffer in all selections because of known interference with gp120 recognition by DARPins^27^. Three to five consecutive rounds of RD selection were conducted to derive pools enriched in target-specific DARPins. One off-rate selection round was performed to select clones with improved affinity. For this purpose, DARPin-ribosome complexes were first allowed to bind to the bead-immobilized target, before a 100- to 1000-fold molar excess of the non-biotinylated target was added as a competitor for 1-2 h, during which time DARPin binders with a fast off-rate are lost. The supernatant was removed, beads washed extensively (10-15 times) and a further RD round without competitor was conducted to amplify the remaining binders^72^.

High-throughput Ribosome Display selection (Selections C-E): Bead coupling was performed as described above. Panning of ternary DARPin-mRNA-ribosome complexes was performed in 1.5 ml microtubes on a KingFisher Flex magnetic particle processor (Thermo Fisher Scientific). Five RD rounds were conducted with decreasing immobilized target concentrations in the first four rounds (250 nM, 125 nM, 50 nM, 5 nM). In the fourth off-rate selection round, a large molar excess of the soluble target (500 nM) was added to select for high-affinity DARPins. In the fifth round (referred to as rescue round)^32,91^, the immobilized target concentration was increased (50 nM) to amplify the remaining high-affinity DARPins.

### DARPin expression and purification

Production of small-scale DARPin batches (1 ml cultures) for primary screens: DARPin pools obtained after the final RD round were cloned into pQE30-based expression vectors with an N-terminal his-tag (selections A and B) or a vector with an N-terminal his-tag and a C-terminal FLAG^®^-tag (selections C, D, and E). *E. coli* XL1 blue bacteria were transformed with the plasmid pools, individual clones picked (94 clones for selections A and B; 190 clones for selections C, D, and E) and cultures were grown in 1 ml deep-well plates to OD_600_ = 0.8. Protein expression was induced with 0.5 mM isopropyl-β-D-1-thiogalactopyranoside (IPTG; Sigma-Aldrich) for 3-4 h. To retrieve DARPins, bacterial pellets were lysed in 50 μl B-PER II (Thermo Fisher Scientific), shaken for 15 min at 1300 rpm, and then incubated without shaking for 50 min at RT. 1 ml TBS/0.1% Tween/500 mM NaCl/0.1% BSA (pH 8) was added and the equilibrated lysate was centrifuged (3000 rpm, 20 min, 4 °C) to remove cell debris. 900 µl of the supernatant was transferred to a new 96-deep well plate and this crude extract was stored at −20 °C and used for the primary screening by ELISA.

For primary neutralization screening (see below), DARPins were purified from 400-900 µl bacterial crude extract in 96-well plates using Ni-NTA-coated magnetic beads (His Mag Sepharose Ni; GE Healthcare). Magnetic beads were equilibrated with PBS and 20 μl of beads were added to each well containing crude extract and incubated for 1 h at 1300 rpm. The supernatant was removed using a MagPlex (Luminex) magnetic plate and beads were washed three times with 300 μl PBS. DARPins were eluted with 150 μl PBS containing 250 mM imidazole (Sigma Aldrich). For selections C, D, and E an additional buffer-exchange step was applied, in which cytotoxic imidazole was removed from DARPin eluates by washing three times with 150 μl PBS on AcroPrep 96 filter plates (Pall, 3 kDa cut-off). Successful purification was monitored for randomly selected clones by SDS-PAGE.

Production of large-scale DARPin batches (200 ml - 1 l cultures): These were produced for all DARPins selected for follow-up experiments. Prior to this purification, the selected clones from selections A and B were also re-cloned into the N-terminal his-tag/C-terminal FLAG^®^-tag vector to allow uniform detection via the Flag tag in forthcoming experiments. DARPins from large-scale production batches were purified as described by Ni-NTA affinity chromatography and by SEC (Size Exclusion Chromatography)^97^. SEC was performed on an ÄKTA Purifier system (GE Healthcare) with a Superdex 200 10/300 GL column (GE Healthcare) and PBS as running buffer. Monomeric fractions were concentrated (Amicon Ultra Centrifugal Filters, Merck Millipore) and stored at −20 °C. DARPin concentrations were calculated by measuring the absorbance at 280 nm wavelength on a nanodrop device (Thermo Fisher Scientific). The specific absorbance coefficients were derived from the amino acid sequences using the Protparam tool on www.expasy.org. All clones selected for detailed characterization were checked by Multi-Angle Light Scattering (MALS) for their oligomerization state. Purified DARPins were separated on a Superdex 200 10/300 GL size exclusion column coupled to a MALS detector at the outlet. UV absorption was measured at 280 nm wavelength and plotted normalized to the peak maximum. DARPins that exhibited a tendency to form dimers or higher-order aggregates were excluded from further analysis.

Broadly neutralizing DARPins (bnDs) were also expressed as bivalent bnD-Fc fusions. To this end, the DARPin sequence was fused to the N-terminus of the coding sequence for the Fc-region of the human IgG1 heavy chain (IGHG1, https://www.ncbi.nlm.nih.gov/nuccore/NC_000014, REGION: 105741473..105743070), preserving the hinge region, and cloned into a pcDNA3.1 expression vector. BnD-Fcs were expressed by transient transfection in 293 F or Expi293F cells and purified from the supernatant on a protein G affinity chromatography column equilibrated in 20 mM phosphate buffer, pH 7.0, and eluted with 100 mM glycine, pH 2.7. The eluate was adjusted to pH 4.0 using 1 M Tris buffer, pH 8.7, concentrated using an Amicon filter unit (10,000 kDa MWCO, Millipore) and immediately purified by SEC on a Superdex 200 10/300 GL Increase (GE Healthcare) column equilibrated in PBS. If immediate purification was not possible, the eluate was first subjected to a buffer exchange (10 mM Tris, 10% maltose adjusted to pH 4 with acetic acid) to prevent aggregation of bnD-Fcs.

### Detection of DARPin and mAb binding to target proteins and peptides by ELISA or Luminex assay

20 nM mono-biotinylated target was immobilized to white high-binding 96- or 384-well microplates (Corning) pre-coated with 66 nM Neutravidin (Thermo Fisher Scientific), either overnight at 4 °C or for 1 h at room temperature (RT), followed by three wash steps with TBST (Tris-buffered saline containing 0.1% Tween 20 (Sigma Aldrich), pH 7.5). Target Env-proteins were either probed unliganded or triggered with 50 nM sCD4 or CD4M47. Serial dilutions of purified DARPins were added to microplates in TBSTB (TBST with 0.5% bovine serum albumin (Sigma Aldrich), pH 7.5). Unbound material was washed off after 1 h with TBST, and bound DARPins were detected via their FLAG®-tag using a mouse anti-FLAG^®^ antibody (Sigma Aldrich, clone M2, Cat#F1804 or Cat#F3165) diluted 1:10’000 in TBSTB. After incubation with alkaline phosphatase-conjugated polyclonal goat anti-mouse IgG (whole molecule) secondary antibody (Sigma Aldrich, Cat#A3562) diluted 1:10’000 in TBSTB, a chemiluminescent substrate (Tropix CDP-star, Thermo Fisher Scientific) was added. The emission of relative light units was recorded on a Dynex Technologies Luminometer. Binding of Fc-bnDs was detected on the same instrument using polyclonal goat anti-human IgG (Fc specific) alkaline phosphatase-conjugated antibody (Sigma-Aldrich, Cat#A9544) diluted 1:60’000 in TBSTB.

For ELISA screen of selections A and B, B-PER lysed, cell-debris-free DARPin crude extracts diluted 1:100 in TBSTB were incubated as described above. Bound DARPins were detected via their His-tag using a monoclonal alkaline phosphatase-conjugated mouse anti-polyhistidine antibody (Qiagen, clone HIS-1, Cat#A5588) diluted 1:8,000 in TBSTB.

For ELISA screen of selections C, D, and E, B-PER lysed, cell-debris-free DARPin crude extracts were diluted 1:100 in PBS / 0.5% BSA. High-binding 384-well microplates (BioGreiner) were coated with 66 nM Neutravidin (Thermo Fisher Scientific) in PBS overnight at 4 °C, followed by three wash steps with PBST (phosphate-buffered saline containing 0.1% Tween 20 (Sigma Aldrich), pH 7.5). Plates were blocked with PBSTB (PBST supplemented with 0.2% (w/v) BSA) for 1 h at 4 °C, followed by three PBST wash steps. Mono-biotinylated target (20 nM) was added for 1 h at RT and residual target removed by three PBST wash steps. DARPin crude-extract dilutions were added and allowed to bind the target for 1 h at 4 °C. FLAG^®^-tagged DARPins were detected with mouse anti-FLAG^®^ antibody (Sigma Aldrich, clone M2, Cat# F3165) diluted 1:10’000 in TBSTB and an alkaline phosphatase-conjugated polyclonal goat anti-mouse IgG (whole molecule) secondary antibody (Sigma Aldrich, Cat#A3562) diluted 1:10’000 in TBSTB. pNPP substrate solution (30 mM para-nitrophenylphosphate, 50 nM NaHCO_3_, 50 nM MgCl_2_) was added, and OD 405 nm and 540 nm was measured on a BIO-TEK Synergy HT Plate Reader.

To measure binding of monoclonal V3-specific antibodies to linear V3 and V3-crown mimetic peptides we used a customized multiplex bead assay using the Luminex technology^©^ as described^98^.

### Env pseudovirus production

Neutralization screens conducted in the frame of this study included sets of five viruses used for the primary screen (Supplementary Fig. 2), an 18-multi clade virus panel, and an extended 40 multi-clade virus panel. The latter has been previously used for breadth and fingerprint analyses of bnAbs and patient plasma^47^. A full list of Env pseudotyped viruses generated with corresponding Env, Genebank entry, clade, and neutralization Tier information is provided in Supplementary Data 1. The SF162P3N_cl8 *env* gene^99^ was a gift from Cecilia Cheng-Mayer (The Aaron Diamond AIDS Research Center, Rockefeller University, NY, USA). Env-pseudotyped viruses were prepared by co-transfection of HEK 293-T cells with plasmids encoding the respective *env* genes and the luciferase reporter HIV vector pNLluc-AM as described^100^.

### Env mutants

V1V2-deleted Env mutants were generated as described previously^9^. A JR-CSF alanine Env mutant library encompassing 123 mutants in gp120 was provided by D. Burton^16,101,102^. All other mutations were generated by site-directed mutagenesis (QuikChange II kit, Agilent, Santa Clara, USA) according to the manufacturer’s instructions. A full list of mutants employed for mutational scanning is depicted in Supplementary Data 7.

### Neutralization assay

The neutralization activity of DARPins and mAbs was evaluated on TZM-bl cells using Env pseudotyped viruses in a 96-well^100^ or 384-well^47^ assay format as described. The input of Env pseudoviruses was chosen to yield virus infectivity corresponding to 5,000–20,000 relative light units (RLU) in the absence of inhibitors as measured on a Dynex MLX luminescence reader (96-well plates). 384-well plates were readout on a Perkin Elmer EnVision Multilabel Reader. The DARPin or Ab concentrations causing a 50% reduction in viral infectivity (inhibitory concentration IC_50_) were calculated by fitting a sigmoid dose-response curve (variable slope) to the data, using Prism versions 7 or 9 (GraphPad Software). If 50% inhibition was not achieved at the highest or lowest inhibitor concentration, a ‘greater than’ or ‘less than’ value was recorded. To control for unspecific effects, all DARPin clones were tested for activity against MuLV pseudovirus.

Primary neutralization screen of DARPins: As the DARPin yield from 1 ml *E. coli* cultures is limited, the primary neutralization screen of DARPin pools was restricted to a single replicate per virus. DARPin preparations from small-scale purifications were diluted 1:6 in a TZM-bl cell culture medium. Viruses probed in these initial neutralization screens were chosen based on target antigens employed in the individual selection and are listed in Supplementary Fig. 2. Percent inhibition in the presence of DARPin compared to mock-treated controls was recorded for each DARPin-virus combination.

Secondary neutralization screens on 18- and 40-virus panels were conducted using purified DARPin preparations with known concentrations. IC_50_ data represent geometric means from one to seven independent experiments.

Neutralization screens with a JR-CSF Env mutant panel: 128 JR-CSF mutant pseudoviruses carrying gp120 point mutations (Supplementary Data 7) were probed for sensitivity to DARPins and selected mAbs in a two-step screening approach to detect resistance-conferring mutants. In a first run, all mutant viruses of the JR-CSF Env panel were screened against all inhibitors. Mutants that passed in this first screen — a pre-set threshold of resistance (5-fold over IC_50_ against wt JR-CSF) against one DARPin — were followed up and retested against all DARPins, yielding in total at least two independent tests (Supplementary Data 8). Mutants that showed no effect against any of the inhibitors were not followed up and hence only tested once.

### Sequence analysis of DARPins and Env clones

Sequencing of DARPins and envelope genes was performed by Sanger sequencing either in-house or at Microsynth AG (Balgach, Switzerland). All envelope sequence data are based on HxB2 numbering.

### Binding of DARPins to cell-expressed Env

DARPin binding to Env expressed on 293 T cells in the presence and absence of two-domain sCD4-183 was done as previously described in^36^. Briefly, 293 T cells were co-transfected with the desired Env-expression plasmid and the pCMV-*rev* expression helper plasmid in a 4:1 ratio and stained 36 h later with biotinylated DARPins. To this end, DARPins were cloned into the pBD002 expression vector in-frame with an N-terminal AviTag and a C-terminal polyhistidine-tag. For expression and biotinylation, *E. coli* XL1 blue bacteria were co-transformed with the AviTag DARPin vector and with the pBirAcm expression plasmid coding for the BirA biotinylation enzyme and selected on agar plates containing 100 µg/ml carbenicillin and 10 µg/ml chloramphenicol. DARPins were then expressed in TYH-medium supplemented with 0.5% glucose. Expression was induced at OD_600_ = 0.8 by adding 50 µM IPTG together with 50 µM D-biotin (Applichem). Biotinylated DARPins were purified as described above on a Ni-NTA column (Qiagen) and by SEC.

Cells were incubated with biotinylated DARPins or DARPin-Fc fusions in the presence or absence of sCD4-183 for 20 min at RT. Bound DARPins were detected via APC-Cy7-conjugated streptavidin (BD Biosciences, San Jose, CA, USA; Cat#554063) diluted 1:500 in FACS-buffer (PBS, 2 mM EDTA, 2% FCS), bound DARPin-Fc fusions, and mAbs via allophycocyanin (APC) conjugated goat anti-Human IgG F(ab’)_2_ (Jackson ImmunoResearch Europe, Ely, UK; Cat#109-136-170) diluted 1:500 in FACS-buffer on a FACSVerse system (BD Biosciences, San Jose, CA, USA) and analyzed using FlowJo 10 software (FlowJo LLC, Ashland, OR, USA). Propidium iodide staining (BD Biosciences, San Jose, CA, USA) diluted 1:500 in FACS-buffer was used to gate for live cells.

### Crystallization and protein structure determination

For crystallization, bnD.1, bnD.2, and bnD.3 were subcloned into plasmid pQiq_H_10__3 C which contains a human rhinovirus 3 C protease-removable 10xhistidine tag. DARPins 63_B7 and 5m3_D12 were produced in pQE30 vectors. DARPins were expressed and Ni-NTA-purified as described above. For bnD.1, bnD.2 and bnD.3, His-tags were removed by 3C protease (2% w/w) cleavage overnight while dialyzing against 50 mM Tris pH 8, 300 mM NaCl. Uncleaved DARPins and 3 C protease were removed by reverse Ni-NTA chromatography. Monomeric DARPin fractions were isolated by size-exclusion chromatography on ÄKTA Pure or Prime systems (GE Healthcare) with HiLoad 16/600 Superdex 75 pg or Superdex 200 10/300 GL columns (GE Healthcare) and 10 mM Tris pH 7.4, 100 mM NaCl (for bnD.1, bnD.2 and bnD.3) or 10 mM HEPES pH 7.4, 150 mM NaCl (for 63_B7 or 5m3_D12) as running buffer. Monomeric fractions were concentrated (Amicon Ultra Centrifugal Filters, Merck Millipore) to 20 mg/ml and supplemented with 1.5–2-fold molar excess of V3 mimetic.

Sparse-matrix screens (Hampton Research, Molecular Dimensions, and Qiagen) in a sitting-drop vapor diffusion format at 4 °C (for bnD.1, bnD.2 and bnD.3) or 20 °C (63_B7 or 5m3_D12) were used to identify initial crystallization conditions; focus screens with pH and precipitant gradients were used to refine initial conditions. Crystals were flash-frozen (liquid N_2_) in mother liquor supplemented with 5–15 % ethylene glycol. Data were collected on beam lines X06DA and X06SA at the Swiss Light Source (Paul Scherrer Institute, Villigen, Switzerland) using a Pilatus or Eiger detector system (Dectris Ltd).

Data were processed using XDS, XSCALE, and XDSCONV^103^ (Version October 15, 2015), 5 % of data were set aside to calculate the *R*_*free*_ value. Initial phases were obtained by molecular replacement using PHASER (2.6.0)^104^ with structures of different N2C and N3C DARPins as search models. Refinement was done using REFMAC5 (version 5.8.0135)^105^, BUSTER (version 2.10.1)^106^, and Phenix-Refine (version 1.11_2567)^107,108^, followed by model building in COOT (version 0.8.8)^109^. We chose a data cut-off according to Karplus and Diederichs^110^ that includes more data than classical approaches. Therefore, the resolutions reported in Supplementary Data 5 are rather high. For the structures with I/sigma<2 the resolutions at a cut-off at I/sigma=2 would be approximately 1.9 Å for 63_B7:V3-IY (PDB ID: 7DNF), 1.67 Å for 63_B7:V3 (PDB ID: 7DNG), 2–2.06 Å for bnD.1:V3-IF (PDB ID: 7B4T), 1.44–1.48 Å for bnD.2:V3-IF (PDB ID: 7B4V J32), and 1.9–1.95 Å for bnD.3:V3-IF (PDB ID: 7B4W) as derived by XSCALE. Structures 7DNF and 7DNG had a relatively low-resolution cut-off (see respective PDB x-ray structure validation report section 1). To retrieve improved cut-offs we employed XSCALE as suggested by Karplus et al.^111^. Cut-offs were applied according to the results from XSCALE at CC half > 10%. In these calculations the CC half of 7DNF and 7DNG are 27.3% and 11.7% at a resolution of 1.78 and 1.42 Å, respectively. With the exception of the resolution cutoff, all values listed in Supplementary Data 5 were generated by the phenix.table_one program and are consistent with the PDB entry. All V3 mimetics could be completely built into different electron densities during refinement. Analysis of binding interfaces was done with LigPlot + (v1.4.2)^112^ and QtPISA (version 2.0.4)^113^. Figures were prepared with PyMOL (The PyMOL Molecular Graphics System, Version 1.8 Schrödinger, LLC).

### Molecular dynamics

Molecular dynamics (MD) simulations were conducted to characterize the conformational space sampled by the gp120 V3 loop. In total, seven systems were investigated: a fully glycosylated and a fully deglycosylated structure of gp120, and five V3 mimetic peptides. The coordinates for the starting structure of gp120 bound to CD4 was extracted from a cryo-electron microscopy structure of gp120 bound to CD4 and the CCR5 co-receptor (PDB ID: 6meo^2^). For the fully glycosylated structure, N-linked mannose-5 glycans (Man5) were modeled onto each sequon, using the python package Glycosylator 1.0^114^. All glycans were removed in the fully deglycosylated structure. The initial coordinates for the V3 mimetics were extracted from previously determined structures^27^. All structures were solvated in a 17 Å padding water box and neutralized with 150 mM NaCl (see Table 1 for details).

**Table 1:** List of performed molecular dynamics simulations.

System	Size of unit cell [Å]	Simulation time [μs]
gp120 glycosylated (mannose 5) bound by sCD4	131 × 130 × 125	0.7
gp120 deglycosylated bound by sCD4	130 × 130 × 124	0.7
V3 linear peptide	62 × 60 × 54	1.2
V3-RF cyclic peptide	60 × 67 × 60	1.2
V3-RY cyclic peptide	54 × 48 × 52	1.2
V3-IF cyclic peptide	54 × 48 × 52	1.2
V3-IY cyclic peptide	50 × 50 × 50	1.2

The simulations were performed with the CHARMM36 force field^115,116^, including CMAP corrections for the protein. TIP3P water parameterization was used to describe the water molecules. The simulations were carried out using the ACEMD 3.0 software^117^. The periodic electrostatic interactions were computed using particle-mesh Ewald (PME) summation with a grid spacing smaller than 1 Å. The constant temperature was imposed by using Langevin dynamics with a damping coefficient of 1.0 ps. The constant pressure of 1 atm was maintained with the Berendsen barostat. The systems were first minimized by 2000 conjugate gradient steps, followed by a free molecular dynamics simulation with a hydrogen mass repartitioning scheme to achieve a timestep of 4 fs. Snapshots from each simulation were extracted at 1 ns time intervals for structural analysis.

### Protein-protein docking

Protein-protein docking was used to investigate the binding of bnD.2, bnD.3 and six V3 targeting nAbs in context of the full gp120 (F425-B4e8 (PDB ID: 2qsc), 2219 (PDB ID: 2b0s), 3074 (PDB ID: 3mlx), 447-542D (PDB ID: 4m1d), DH753 (PDB ID: 6mnr), PGT135 (PDB ID: 4jm2)).

For each structure, the co-crystallized V3 peptide was aligned to the different conformations sampled by the V3 loop during the MD simulation of the fully glycosylated gp120. The frame with the smallest root-mean-square deviation (RMSD) between the peptide and the V3 loop was extracted and used as the initial pose for the docking procedure in the context of an open (CD4 and mAb 17b-bound) Env trimer structure (PDB ID: 5vn3^11^). In addition, bnD.3 was also docked to the partially open trimer (PDB ID: 5vn8^11^).

The RosettaDock (2018.33.60351) procedure was used for the protein-protein docking^118^. For each structure, 700 poses were generated. The structure with the lowest energy and an RMSD to the initial pose smaller than 5 Å were considered as successful docking.

### Swiss 4.5 K Screen study population and ethics information

In the current study, 4,281 plasma samples from HIV-1 infected individuals included in the Swiss 4.5 K Screen^13,47^ were re-analyzed for binding to V3-crown peptides. A detailed description of sample/patient selection and study design of the Swiss 4.5 K Screen has been described previously^47^. All analyzed plasma samples were derived from specimens stored in the biobanks of the Swiss HIV Cohort Study (SHCS) and the Zurich Primary HIV Infection Study (ZPHI). The Swiss HIV Cohort Study (SHCS) is a prospective, nationwide, longitudinal, non-interventional, observational, clinic-based cohort with semi-annual visits and blood collections, enrolling all HIV-infected adults living in Switzerland^119^. The SHCS, founded in 1988, is highly representative of the HIV epidemiology in Switzerland as it includes an estimated 53% of all HIV cases diagnosed in Switzerland since the onset of the epidemic, 72% of all patients receiving ART in Switzerland, and 69% of the nationwide registered AIDS cases^119,120^. The SHCS is registered under the Swiss National Science longitudinal platform: http://www.snf.ch/en/funding/programmes/longitudinal-studies/Pages/default.aspx#Currently%20supported%20longitudinal%20studies. Detailed information on the study is openly available on http://www.shcs.ch.

The Zurich Primary HIV Infection Study (ZPHI) is an ongoing, observational, non-randomized, single-center cohort founded in 2002 that specifically enrolls patients with documented acute or recent primary HIV-1 infection (www.clinicaltrials.gov; ID NCT00537966)^121^.

The SHCS and the ZPHI have been approved by the ethics committee of the participating institutions (Kantonale Ethikkommission Bern, Ethikkommission des Kantons St. Gallen, Comité départemental d’éthique des spécialités médicales et de médicine communautaire et de premier recours, Kantonale Ethikkommission Zürich, Repubblica e Cantone Ticino - Comitato Ethico Cantonale, Commission cantonale d’éthique de la recherche sur l'être humain, Ethikkommission beider Basel for the SHCS and Kantonale Ethikkommission Zürich for the ZPHI) and written informed consent had been obtained from all participants.

### Profiling of the Swiss 4.5 K cohort for V3-crown specific HIV-1 binding antibodies

We used a customized multiplex bead assay using the Luminex^©^ technology to measure the plasma IgG1 binding antibody activity to the four V3-crown mimetic peptides and mimetics in 4,281 individuals included in the Swiss 4.5 K cohort as described^13,98^. In addition to the reactivities measured here we utilized binding data established for three linear V3 peptides (based on BG505, MN, and JR-FL sequences), JR-FLgp120, BG505gp140, and trimeric BG505-SOSIP determined for the same samples in a previous study from our group^13^. Based on the high prevalence of IgG1 antibodies to V3 and the optimal plasma dilution for the binding assay that we previously established^13^, we assessed V3-binding IgG1 antibody profiles at a fixed plasma dilution of 1:6500. Otherwise, the assay was conducted as described^13^. Recorded binding intensities were transformed into relative binding data by performing ordinal ranking of a plasma sample relative to all samples tested on the same day (ranging between 122 to 354 samples tested per day) to rule out intra- and inter-assay variability as described^13,98^.

### Dissection of neutralization, host, viral and disease parameters that are linked with V3-IgG1 binding

Data on eight host, viral, and disease parameters of Swiss 4.5 K individuals was available from our prior studies on this cohort^13,47^. Neutralization breadth was used as a categorical response variable. Patients were defined to have neutralization breadth if their plasma reached cross-, broad- or elite-neutralization activity score as determined in^47,122^. Patients with no or weak neutralization activity scores were categorized as having no neutralization.

We investigated the association of these parameters with V3-IgG1 reactivity by univariable and multivariable linear regression (using Python 2.7 and its library statsmodels 0.8.0-3) as described^13^. Log10 viral load and CD4 level (both measured at the time of sampling); viral *pol* diversity^123^ and infection length were included as continuous variables. The remaining four factors were used as categorical variables and analyzed in relation to the reference category (sex: reference male; mode of transmission: reference men having sex with men (MSM); ethnicity: reference White; HIV-1 clade: reference clade B, neutralization breadth: reference no neutralization).

The dimensionality reduction method t-SNE^124^ was used to display the reactivity with V3 peptides across the entire cohort in Fig. 6b and Supplementary Fig. 15a. Supplementary Fig. 15b utilizes the same t-SNE plot to visualize the similarity of V3 peptide-binding responses in the top 105 bnAb plasma samples identified in the Swiss 4.5 K Screen^47^. Dominant bnAb specificities in these 105 bnAb plasma samples were previously determined by neutralization fingerprinting^47^. The location of bnAb plasma samples with distinct specificities is visualized on the t-SNE map.

### Statistics

Statistical analyses were performed in Python 3.7 using the packages scipy.stats, statsmodels, and tsne.

The dimensionality reduction method t-SNE was used to display the plasma samples in two dimensions. As a result of the large cohort size, we used the well-established Barnes-Hut-SNE approximation (with 1000 iterations and parameters theta = 0.5 and perplexity = 200) instead of the exact t-SNE method^124^.

All reported statistical analyses about the V3 reactivity in the Swiss 4.5 K cohort are of explorative, descriptive nature. We therefore opted, by default, not to formally adjust for multiple testing since false positives are less of a problem in explorative studies than false negatives. In addition, owing to the large size of our cohort, almost all the associations we focus on exhibit a very low p-value, even lower than a Bonferroni-corrected p-value.

### Reporting summary

Further information on research design is available in the Nature Research Reporting Summary linked to this article.

## Supplementary information

Supplementary Information

Description of Additional Supplementary Files

Supplementary Data 1

Supplementary Data 2

Supplementary Data 3

Supplementary Data 4

Supplementary Data 5

Supplementary Data 6

Supplementary Data 7

Supplementary Data 8

Supplementary Data 9

Supplementary Data 10

Supplementary Data 11

Supplementary Data 12

Reporting Summary

## Data Availability

The structural data on DARPin:V3 complexes generated in this study (Fig. 3 and Supplementary Fig. 9) have been deposited in the Protein Data Bank (PDB) database under accession codes 7DNE (5m3_D12:V3-IY), 7DNF (63_B7:V3-IY), 7DNG (63_B7:V3), 7B4T (bnD.1:V3-IF), 7B4U J06 and 7B4V J32 (bnD.2:V3-IF) and 7B4W (bnD.3:V3-IF). Supplementary Data 5 with corresponding data collection and refinement statistics is included in the source data file. Other publicly available datasets from the PDB used in this study (Figs. 3 and 4, Supplementary Figs 3, 10, 11, and 14) are accessible under PDB IDs 6MEO (CCR5:gp120:sCD4), 5VN8 (b12 Fab:B41-SOSIP trimer), 3GHE (537-10D Fab:V3), 2QSC (F425-B4e8 Fab:V3), 2B0S (2219 Fab:V3), 3MLX (3074 Fab:V3), 4M1D and 2ESX (447-52D Fab:V3), 6MNR (DH753 Fab:V3), 4JM2 (PGT135 Fab:gp120:17b Fab:sCD4). Source Data is provided in Supplementary Data. Additional source data related to Rusert et al. ^47^, and Kadelka et al.^13^, can be found online under 10.1038/nm.4187 and 10.1084/jem.20180246, respectively.
