# Supplementary material for: Distinct conformations of the HIV-1 V3 loop crown are targetable for broad neutralization
Source: Nat Commun. 2021 Nov 18;12:6705. doi: 10.1038/s41467-021-27075-0 (PMC8602657; doi:10.1038/s41467-021-27075-0)
Supplement: Supplementary file 1 — Supplementary Information [file 41467_2021_27075_MOESM1_ESM.pdf]

Supplementary Information to the Article entiteled:

Distinct conformations of the HIV-1 V3 loop crown are targetable for broad neutralization

Nikolas Friedrich[1$], Emanuel Stiegeler[1$], Matthias Glögl[1$], Thomas Lemmin[1,2], Simon Hansen[3], Claus Kadelka[4], Yufan Wu[3], Patrick Ernst[3], Liridona Maliqi[1], Caio Foulkes[1], Mylène Morin[5], Mustafa Eroglu[1], Thomas Liechti[1], Branislav Ivan[1], Thomas Reinberg[3], Jonas V. Schaefer[3], Umut Karakus[1], Stephan Ursprung[1], Axel Mann[1], Peter Rusert[1], Roger D. Kouyos[1,6], John A. Robinson[5], Huldrych F. Günthard[1,6], Andreas Plückthun[3], Alexandra Trkola[1*]

[1] Institute of Medical Virology, University of Zurich (UZH), Zurich, Switzerland

[2] Department of Computer Science, ETH Zurich, Zurich, Switzerland

[3] Department of Biochemistry, University of Zurich (UZH), Zurich, Switzerland

[4] Department of Mathematics, Iowa State University, Ames, IA, USA

[5] Department of Chemistry, University of Zurich (UZH), Zurich, Switzerland

[6] Division of Infectious Diseases and Hospital Epidemiology, University Hospital Zurich (USZ), Zurich, Switzerland

[$] These authors contributed equally.

[*]correspondence: trkola.alexandra@virology.uzh.ch

DARPin Design

Designed Ankyrin Repeat Proteins (DARPins) are repeat proteins consisting of two capping repeats (N- and C - cap) and internal ankyrin repeats with randomized residues [A,B]. The internal repeats can be varied in number (typically 2-4, often 3), and this format is then called N3C. The internal repeats are based on a structural consensus design derived from all natural ankyrin repeat sequences [A], and the potential target binding residues are randomized in each internal repeat to obtain a synthetic library [B].

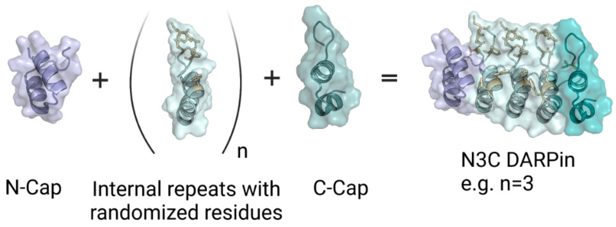

DARPin Protein Properties

This design creates a rigid interaction surface with extreme diversity (limited only by the physical library size), leading to very stable proteins that can bind to targets with very high affinity and specificity, equivalent to antibodies [C]. DARPins show high thermodynamic stability, resistance to denaturants, small size, and a rigid binding mode that allows capture of specific protein conformations [D].

DARPin biotechnological characteristics and applications

DARPins contain no cysteines and fold very efficiently, they can be produced in the cytoplasm of *E. coli* fast and economically, including at large scales . For all these reasons, they have become useful binding molecules for research, diagnostics and therapy [E,F]. DARPins can be linked to homomeric and heteromeric multimers in many formats and fused in many ways [C,E].

DARPin Libraries

To obtain specific binders, different DARPin libraries have been created [B,C]. They can be selected by different selection systems, most typically by ribosome display [G]. In this process, the library is in the form of a PCR amplicon containing a promoter, the open reading frame but no stop codon [H]. It is transcribed and translated in vitro. The DARPin, emerging out of the ribosome and folding to its native form, still remains connected to the tRNA via a C-terminal tether in the ribosomal tunnel. Thus, DARPin and mRNA stay connected via the ribosome, and the complexity of the library is essentially only limited by the number of ribosomes [G,H].

Ribosome Display

Ribosome display permits the direct use of error prone PCR in the selection and thus incorporates the affinity maturation directly in the selection [I]. Thereby, affinity can be increased, and specificity can be tuned by counterselections [D].

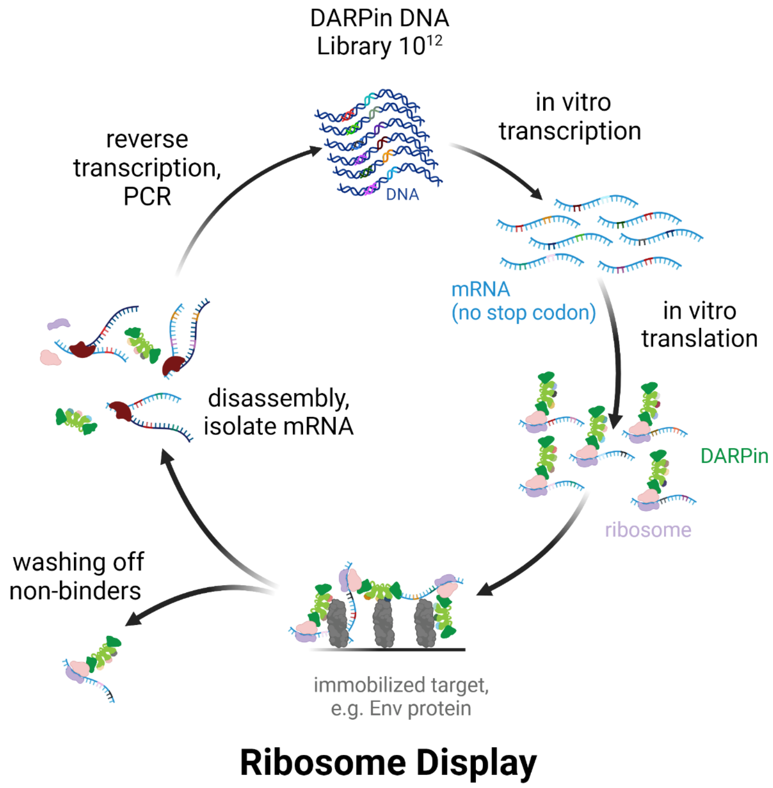

DARPins as therapeutics in the clinic

DARPins have been developed as therapeutic agents for diverse disease settings and are in late-stage clinical trials [J]. For clinical settings where a long half-live is desired, diverse options exist to engineer DARPins with fusion to Fc, serum albumin [K], HSA binding domains [L], or unstructured polymers [M].

References

A Binz H. K. et al., J. Mol. Biol. 332, 489-503 (2003).
B Binz H. K. et al., Nat. Biotechnol. 22, 575-582 (2004).
C Plückthun A., Annu. Rev. Pharmacol. Toxicol. 55, 489-511 (2015).
D Kummer L. et al., Proc. Natl. Acad. Sci. U. S. A. 109, E2248-2257 (2012).
E Tamaskovic R. et al., Methods in Enzymology 503, 101-134 (2012).
F Zahnd C. et al., Cancer Research 70, 1595-1605 (2010).
G Plückthun A. Methods Mol. Biol. 805, 3-28 (2012).
H Zahnd C. et al., Nat. Methods 4, 269-279 (2007).
I Zahnd C. et al. J. Mol. Biol. 369, 1015-1028 (2007).
J clinicaltrials.gov clinical trials identifiers NCT04834856, NCT04828161, NCT04870164, NCT04501978, NCT04049903, NCT03335852, NCT03539549, NCT02859766, NCT02462486, NCT02462928, NCT02181517, NCT02181504, NCT02186119
K Merten H. et al. Eur. J. Pharm. Biopharm.,167, 104-113 (2021).
L Steiner D. et al., Protein Eng Des Sel. 30, 583-591(2017).
M Brandl F. et al., J. Control. Release 307, 379-392 (2019).

Supplementary Fig. 1: DARPin and Ribosome Display Technology

Background information on the Designed Ankyrin Repeat Protein (DARPin) Technology, the Ribosome Display selection procedure applied in the current study to derive target-specific DARPins from synthetic, high-diversity DARPin libraries and the development of DARPins as therapeutics.

a

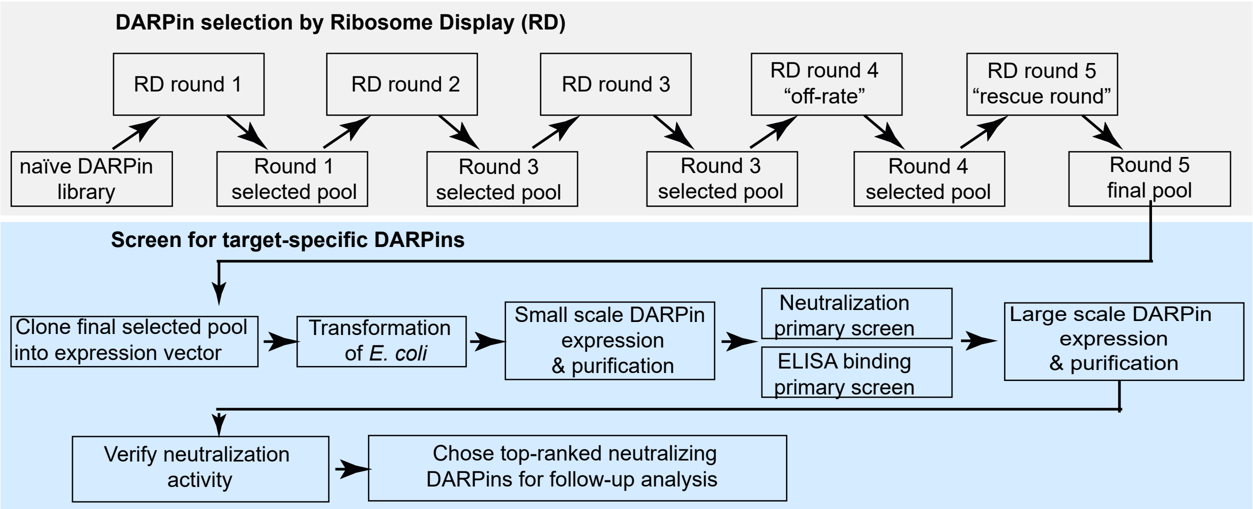

b

Summary of selection strategies pursued to obtain V3-specific and/or Env-reactive DARPins

		Selection A	Selection B	Selection C	Selection D	Selection E
Ribosome Display (RD)	starting DARPin library	1st generation	1st generation	2nd generation	2nd generation + loop DARPins	2nd generation + loop DARPins
	round 1 panning target					
	round 2 panning target					
	round 3 panning target					
	round 3-plus panning target		na	na	na	na
	round 4 panning target					
	round 4 off rate competitor					
	round 5 panning target					
Primary ELISA screen of RD round 5 pools	DARPin clones screened (n)	n=94	n=94	n=190	n=190	n=190
	target 1 (% of total)	95%	0%	10%	8%	1%
	target 2 (% of total)	1%	1%	1%	0%	na
	target 1 and 2 (% of total)	0%	0%	83%	82%	na
	no target binding (% of total)	4%	99%	6%	10%	99%
	binding activity					
Primary Virus neutralization screen of RD round 5 pools	DARPin clones screened (n)	n=94	n=94	n=190	n=190	n=1
	virus strains tested					
	neutralizing DARPins	6%	7%	19%	61%	n=1
	non-neutralizing DARPins	94%	93%	81%	39%	n=0
	clones with neutralizing activity					
Neutralising DARPins selected for follow-up analyses		63_B7	SE4, SB11	bnD.1	bnD.2	bnD.3

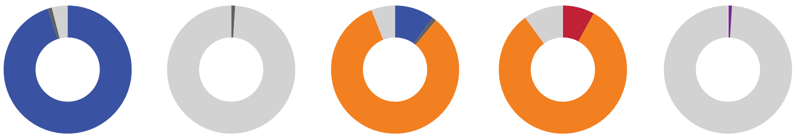

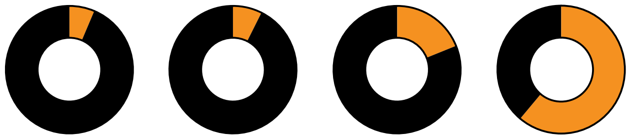

peptide target
V3-IY (MN)
JR-FLgp120+CD4M47
ZM651gp120+sCD4
BG505_SOSIP
JR-FLgp120ΔV1V2
target 1 and 2 binding
no target binding

virus strain (subtype)
SF162 (B)
JR-FL (B)
JR-FL D197N (B)
JR-FL D197NΔV1V2 (B)
MG505 (A)
BG505 T332N (A)
CAP88_1mo.c17 (C)
ZM214 (C)

Supplementary Fig. 2: Overview DARPin panning and screening

a Workflow of ribosome display and screening as used in this study. **b** Summary of five selections (A-E) pursued to obtain V3-specific and/or Env-reactive DARPins. Strategies differ by the DARPin library used for Ribosome Display (RD) and the selection targets employed in successive RD panning rounds. Targets were alternated in some selections to achieve cross-reactivity. Target 1 and target 2 refers to the ones used in each selection according to the color code. Primary binding and neutralization screens were adjusted to match selection targets. DARPins binding in ELISA three-fold over background were categorized as specific binders. DARPins neutralizing at least one of the tested Env-pseudotyped viruses were categorized as neutralizing. The six best neutralizing DARPins were chosen for follow-up analyses (bottom row).

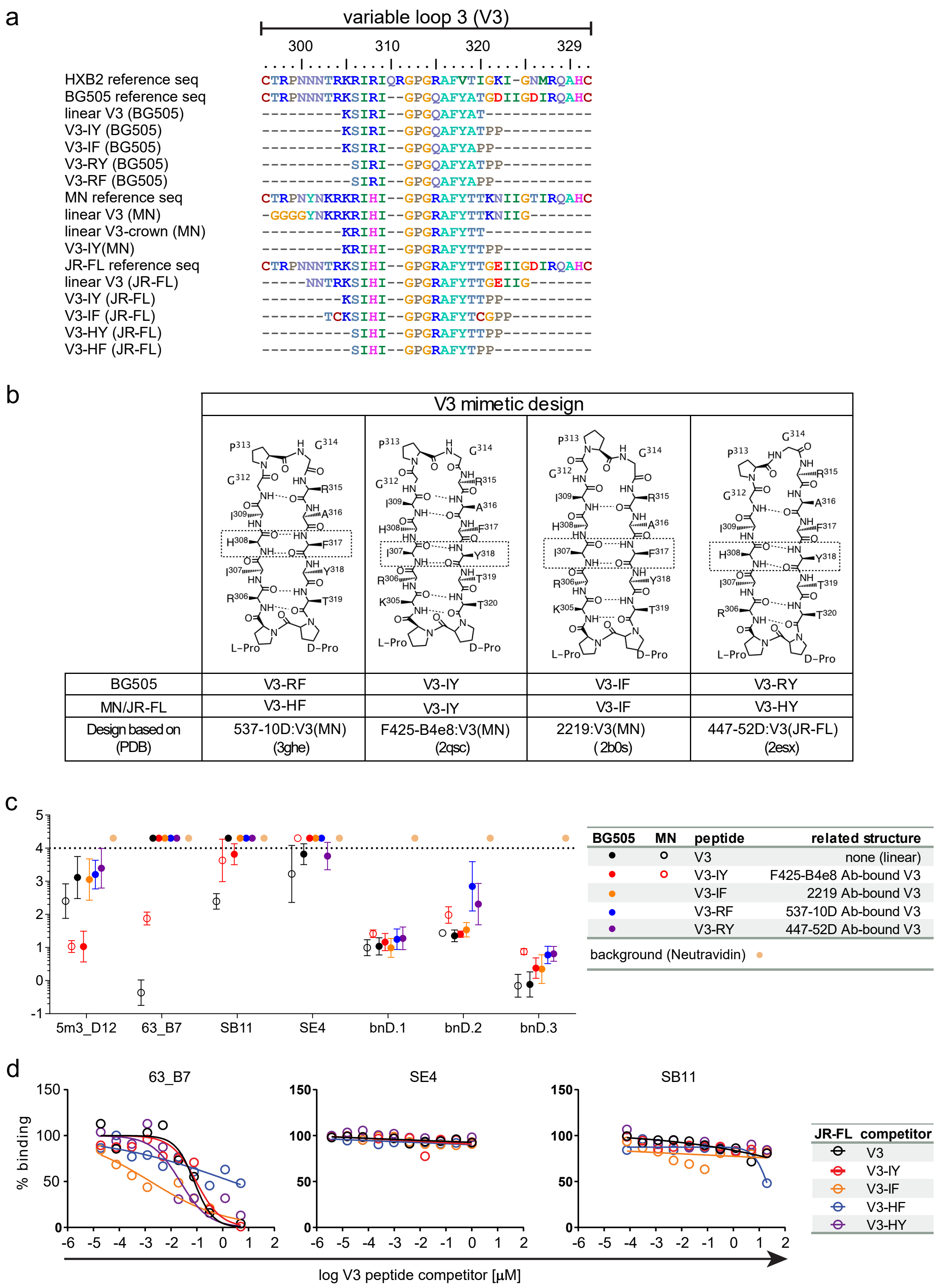

Supplementary Fig. 3: V3 mimetic design and DARPin binding

a Sequence alignment of V3 regions from four different HIV-1 strains (clade B strains HxB2, MN and JR-FL, and clade A strain BG505) and corresponding linear and cyclic structurally constrained mimetic peptides used in the present study. The cyclic structurally constrained peptides contain the sequence PP, as shown in **b**. **b** Chemical structure of the cyclic V3 mimetic design (based on the sequence of strain MN). The construction of the mimetics has been described in Riedel et al. [39]. The mimetics were designed to resemble the structure of linear V3 peptides in complex with the indicated V3 mAbs. The cyclic structurally constrained mimetics are built on a D-Pro/L-Pro template to achieve inter-strand hydrogen bond pairing with four different registers (IY, IF, RY/HY and RF/HF) that capture the structure of V3 bound by the four respective mAbs as indicated in the figure. Note that the V3-HY and V3-HF mimetics (based on strains JR-FL/MN) are equivalent in inter-strand hydrogen bonding to V3-RY and V3-RF mimetics (based on strain BG505), respectively. The naming differs due to a polymorphism in position 308 (H/R). **c** Binding of DARPins to biotinylated V3 mimetics immobilized via neutravidin in ELISA. Data are geometric means and geometric SDs of EC_{50} values from independent replicates (n=3). The maximum concentration probed is indicated by a dashed line. If no EC_{50} could be determined, the respective symbol is placed above this line. Source data are provided as a Source Data file. **d** Competition binding ELISA to probe for V3-crown conformational preferences of DARPins. DARPin binding to immobilized Env targets was measured at a concentration just below reaching saturation. Env targets were chosen to maximize the dynamic binding range for the individual DARPins (63_B7: JR-FLgp120+sCD4; SE4: JR-FLgp120ΔV1V2+M47; SB11: JR-FLgp120+M47). Each DARPin/Env pair was competed with increasing concentrations of the JR-FL V3 peptides indicated in the legend (V3-HF and V3-HY are equivalent to V3-RF and V3-RY, as explained in **b**).

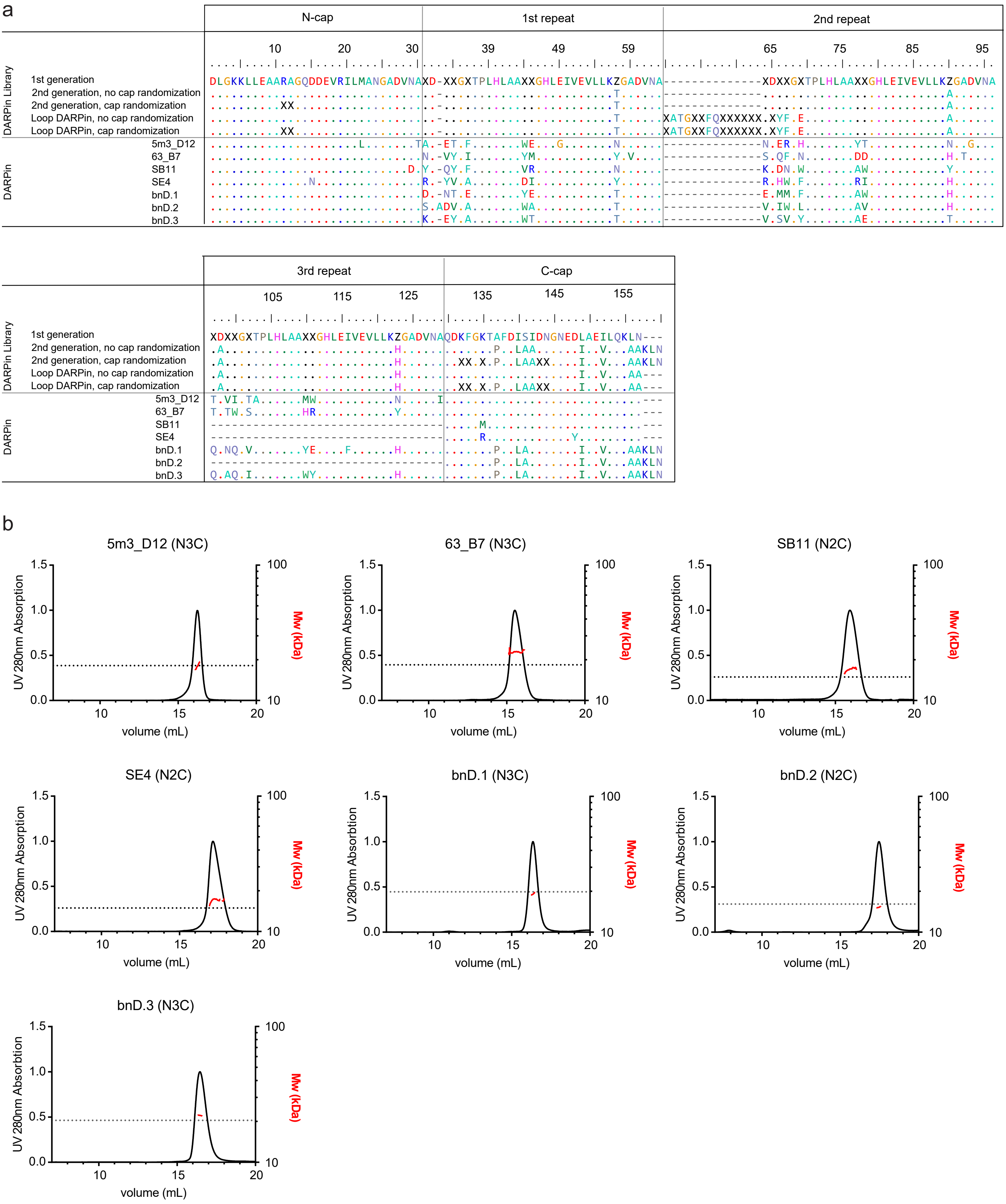

Supplementary Fig. 4: DARPin sequences and analysis by Multi-Angle-Light-Scatter (MALS)

a Amino acid sequence alignment of the selected V3-crown specific DARPin clones and consensus sequences of DARPin libraries used for Ribosome Display selections (the second generation and loop DARPin libraries each comprise clones with and without cap randomization). DARPins consist of flanking N-terminal and C-terminal capping repeats, and internal randomized repeats. Randomized position X allows for any amino acid except Cys, Pro and Gly, while position Z allows only His, Asn, Tyr). Most DARPins selected contain 3 internal repeats (referred to as N3C DARPins), except SB11, SE4 and bnD.2 which contain only two internal repeats (referred to as N2C DARPins). **b** MALS analysis of selected DARPins (N2C- or N3C-type according to **a**, indicated in parentheses), shown in red (right y-axis). UV absorption normalized to the maximum is depicted. Dotted lines indicate the theoretical molecular weight of monomeric DARPin forms as determined from their amino acid sequences.

a

clade	virus strain	variable loop 3 (V3) (300 … 310 … 320 … 328)
B	HxB2 (reference sequence)	CTRPNNNTRKRIRIQRGPGRAFVTIGKI-G-NMRQAHC
A	BG505_W6M_C2_T332N	CTRPNNNTRKSIRI--GPGQAFYATGDIIG-DIRQAHC
A	KER2018.11	CARPSNNTRKSVHI--GPGQAFYATGAITG-DIRQAYC
A	MG505.W0M.ENV.A2	CTRPNNNTRKSIRI--GPGQAFYATGDIIG-DIRQAHC
A	Q23_17	CIRPNNNTRKSIRI--GPGQAFYATGDIIG-DIRQAHC
A	Q769_H5	CIRPGNNTRKSIHL--GPGKVFYAT-NIIG-DIRKAHC
A	Q842_D12	CTRPNNNTRKSIHI--GPGQAFYATGDIIG-DIRQAHC
B	ZEnv16_1202_7	CTRPGNNTRRSIHI--GPGRAFY-TGEIIG-DIRQAHC
B	ZEnv07_0504_15	CIRPGNNTRKSITI--GPGRAFYTTGEVIG-NIRQAHC
B	ZEnv91_0505_12	CTRPGNNIRKSIPI--GPGRAFFATGDIIG-DIRQAHC
B	BaL_26	CTRPNNNTRKSIHI--GPGRAFYTTGEIIG-DIRQAHC
B	JR-CSF	CTRPSNNTRKSIHI--GPGRAFYTTGEIIG-DIRQAHC
B	JR-FL	CTRPNNNTRKSIHI--GPGRAFYTTGEIIG-DIRQAHC
B	NAB5pre_cl_1	CTRPSNNTRRSITI--GPGRAFYATGDIIG-DIRKAHC
B	NAB9pre_cl_106	CARPNNNTKRSVNI--VPGRAIYTT-DIIG-DIRQAHC
B	PVO.04	CTRPNNNTRKSISI--GPGRAFYATGDIIG-DIRQAHC
B	QH0692, clone 42	CTRPGNNTRKSIHI--GPGRAFYATGDIIG-DIRQAHC
B	REJO4541 clone 67	CTRPNNNTRKSIHI--APGRAFYATGEIIG-DIRKAYC
B	RHPA4259 clone 7	CTRHNNNTRKSINI--GPGRAFYATGKIIG-DIRQAHC
B	TRO clone 11	CTRPNNNTRRSIHI--GPGRAFYATGDIIG-DIRQAHC
B	WITO4160 clone 33	CTRPGNNTRRSINI--GPGRAFYATGAIIG-DIRKAHC
C	25925_2_22	CTRPNNNTRKSIRI--GPGQTFYATGAIIG-NIREAHC
C	CAP45.2.00.G3	CRRPNNNTRKSIRI--GPGQAFYATNDIIG-DIRQAHC
C	CAP88_6mo.c10	CIRPSNNTRKSIRI--GPGQAFYATNAITG-DIRQAHC
C	Du156.12	CTRPNNNTRKSVRI--GPGQTFYATGDIIG-DIRQAHC
C	DU422.1	CTRPNNNTRKSVRI--GPGQTFYATGEIIG-DIREAHC
C	ZM106F.PB9	CTRPNNNTRKSIRI--GPGQTFYATGEIIG-DIRKAYC
C	ZM214M.PL15	CMRPGNNTRRSVRI--GPGQTFYATGEIIG-DIRQAHC
C	ZM233M.PB6	CTRPNNNTRKSIRI--GPGQSFYATGEIVG-NIREAHC
C	ZM249M.PL1	CTRPNNNTRKSIRI--GPGQTFYATGEIIG-KIREAHC
C	ZM53_12	CTRPGNNTRKSIRI--GPGQAFFATTNIIG-DIRQAYC
G	T252_7	CIRPSNNTRRSISS--GPGQVFYTTGEIIGGRVRQAHC
G	NAB13pre_cl_9	CTRLNNNTRKSITF--GPGQAFYATGQIIG-NIRQAHC
G	X2088_c9	CIRPNNNTRRSIHL--GPGRAFYAT-DIIG-DIRQAHC
CRF01_AE	ZEnv32_0111_5	CTRPFNNTRTSITI--GPGQVLYRTGDITG-DIRKAYC
CRF01_AE	ZEnv92_1008_8	CTRPSNNTRTGVHI--GPGQVFYRTGEIIG-DIRKAHC
CRF01_AE	C1080.c3	CTRPSNNTRTSVTI--GPGQVFYRTGDIIG-DIRKAYC
CRF01_AE	CNE5	CTRPYNNTRKSITI--GPGQVFYRQGEIIG-DIRKAYC
CRF01_AE	CNE59	CIRPSTNTRTSVRI--GPGQVFYRTGDIIG-DIRKAYC
CRF02_AG	T250_4	CTRPNNNTRKSIRI--GPGQTFYATGDIIG-NIRKAYC
CRF07_BC	CNE40	CERPNNNTRKSIRI--GPGQTFYATGDIIG-NIREAHC

b

clade	virus strain	variable loop 3 (V3) (300 … 310 … 320 … 329)
B	HxB2 (reference sequence)	CTRPNNNTRKRIRIQRGPGRAFVTIGKI-GNMRQAHC
A	BG505 T332N	CTRPNNNTRKSIRI--GPGQAFYATGDIIGDIRQAHC
B	SF162	CTRPNNNTRKSITI--GPGRAFYATGDIIGDIRQAHC
B	Bal.26	CTRPNNNTRKSIHI--GPGRAFYTTGEIIGDIRQAHC
B	JR-FL	CTRPNNNTRKSIHI--GPGRAFYTTGEIIGDIRQAHC
B	RHPA4259 clone 7	CTRHNNNTRKSINI--GPGRAFYATGKIIGDIRQAHC
B	ZEnv16_0306_14	CTRPSNNTRRSIHI--GPGKAFY-TGGIIGDIRQAHC
B	AC 10.0.29	CIRPNNNTRKGIHI--GPGRAFYTTGDIIGDIRQAHC
B	REJO4541 clone 67	CTRPNNNTRKSIHI--APGRAFYATGEIIGDIRKAYC
B	SF162P3N_cl8	CTRPNNNTRKSIHI--GPGRAFYATGDIIGDIRQAHC
C	CAP88_6mo.c10	CIRPSNNTRKSIRI--GPGQAFYATNAITGDIRQAHC
C	ZM214	CMRPGNNTRRSVRI--GPGQTFYATGEIIGDIRQAHC

Supplementary Fig. 5: V3 sequence alignment

V3 sequence alignment (HxB2 numbering) of virus panels used. HxB2 is shown as reference (not part of the panels). **a** Alignment of V3 amino acid sequences from strains of the cross-clade 40 virus neutralization panel. **b** Alignment of V3 amino acid sequences from Envs of which V1V2-deleted versions were used in this study.

a

		Most frequent amino acids (group M)*					
HxB2 aa	HxB2#	% 1st	1st aa	% 2nd	2nd aa	% 3rd	3rd aa
C	296	99.9	C	0.1	Y	0.04	R
T	297	79.8	T	12.2	I	2.8	V
R	298	99.8	R	0.1	K	0.1	G
P	299	97.6	P	0.9	L	0.5	T
N	300	60.4	N	16.6	G	16.4	S
N	301	96.6	N	0.8	K	0.6	T
N	302	96.9	N	1.1	K	0.5	Y
T	303	95.1	T	1.9	I	1.1	K
R	304	95.3	R	1.3	I	1.3	S
K	305	67.2	K	12.9	T	12.6	R
R	306	80.0	S	14.3	G	4.0	R
I	307	71.6	I	19.4	V	3.9	M
R	308	41.6	R	31.4	H	6.7	P
I	309	79.2	I	9.3	M	7.9	L
Q	310	99.1	-	0.6	G	0.2	H
R	311	99.1	-	0.5	I	0.1	R
G	312	96.6	G	2.8	A	0.2	V
P	313	94.9	P	1.3	Q	1.1	W
G	314	99.5	G	0.1	R	0.1	W/L
R	315	55.6	Q	32.9	R	5.5	K
A	316	52.3	A	29.7	T	13.1	V
F	317	82.0	F	6.4	L	5.4	W
V	318	86.5	Y	9.6	F	1.7	H
T	319	67.4	A	18.6	T	7.6	R
I	320	86.7	T	3.0	N	2.5	A
G	321	77.9	G	5.3	N	3.7	D
K	322	45.0	D	22.1	E	7.4	A
I	323	92.5	I	6.5	V	0.2	M
-	323Ins	86.7	I	6.7	T	4.4	V
G	324	99.2	G	0.3	R	0.1	D
N	325	80.0	D	17.4	N	0.8	E
M	326	97.4	I	0.7	P	0.7	T
R	327	98.3	R	1.3	K	0.1	G
Q	328	68.5	Q	20.8	K	4.6	R
A	329	99.6	A	0.1	T	0.1	S
H	330	70.1	H	26.2	Y	1.6	F
C	331	99.8	C	0.1	R	0.04	X

b

	Most frequent amino acids (40-virus-panel)					
HxB2#	% 1st	1st aa	% 2nd	2nd aa	% 3rd	3rd aa
296	100.0	C	0.0		0.0	
297	70.0	T	17.5	I	5.0	A
298	100.0	R	0.0		0.0	
299	95.0	P	2.5	L	2.5	H
300	55.0	N	20.0	S	20.0	G
301	97.5	N	2.5	T	0.0	
302	100.0	N	0.0		0.0	
303	97.5	T	2.5	I	0.0	
304	97.5	R	2.5	K	0.0	
305	70.0	K	20.0	R	10.0	T
306	97.5	S	2.5	G	0.0	
307	80.0	I	20.0	V	0.0	
308	40.0	R	30.0	H	15.0	T
309	90.0	I	5.0	L	2.5	F/S
310	100.0	-	0.0		0.0	
311	100.0	-	0.0		0.0	
312	95.0	G	2.5	A	2.5	V
313	100.0	P	0.0		0.0	
314	100.0	G	0.0		0.0	
315	60.0	Q	37.5	R	2.5	K
316	60.0	A	20.0	T	17.5	V
317	95.0	F	2.5	I	2.5	L
318	95.0	Y	5.0	F	0.0	
319	70.0	A	15.0	T	12.5	R
320	97.5	T	2.5	Q	0.0	
321	85.0	G	7.5	-	5.0	N
322	45.0	D	35.0	E	10.0	A
323	97.5	I	2.5	V	0.0	
323Ins	90.0	I	7.5	T	2.5	V
324	100.0	G	0.0		0.0	
325	80.0	D	15.0	N	2.5	K/R
326	97.5	I	2.5	V	0.0	
327	100.0	R	0.0		0.0	
328	60.0	Q	27.5	K	12.5	E
329	100.0	A	0.0		0.0	
330	77.5	H	22.5	Y	0.0	
331	100.0	C	0.0		0.0	

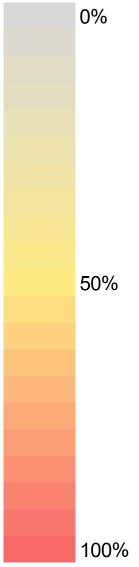

Supplementary Fig. 6: Amino acid sequence conservation in the variable loop 3

The amino acid frequency was determined for all positions (HxB2 numbering) in V3 **a** across group M (*based on 5022 Env sequences not containing premature STOP codons or frameshifts downloaded from the Los Alamos National Library HIV-1 Env sequence collection on March 1st, 2019) and **b** for the multi-clade 40-virus-panel. The most frequent amino acid (% 1st, 1st aa), the second most frequent amino acid (% 2nd, 2nd aa) and the third most frequent amino acid (% 3rd, 3rd aa) are depicted. Note that the V3 consensus sequences of group M and of the 40-virus panel are identical to each other and to the BG505 V3 sequence.

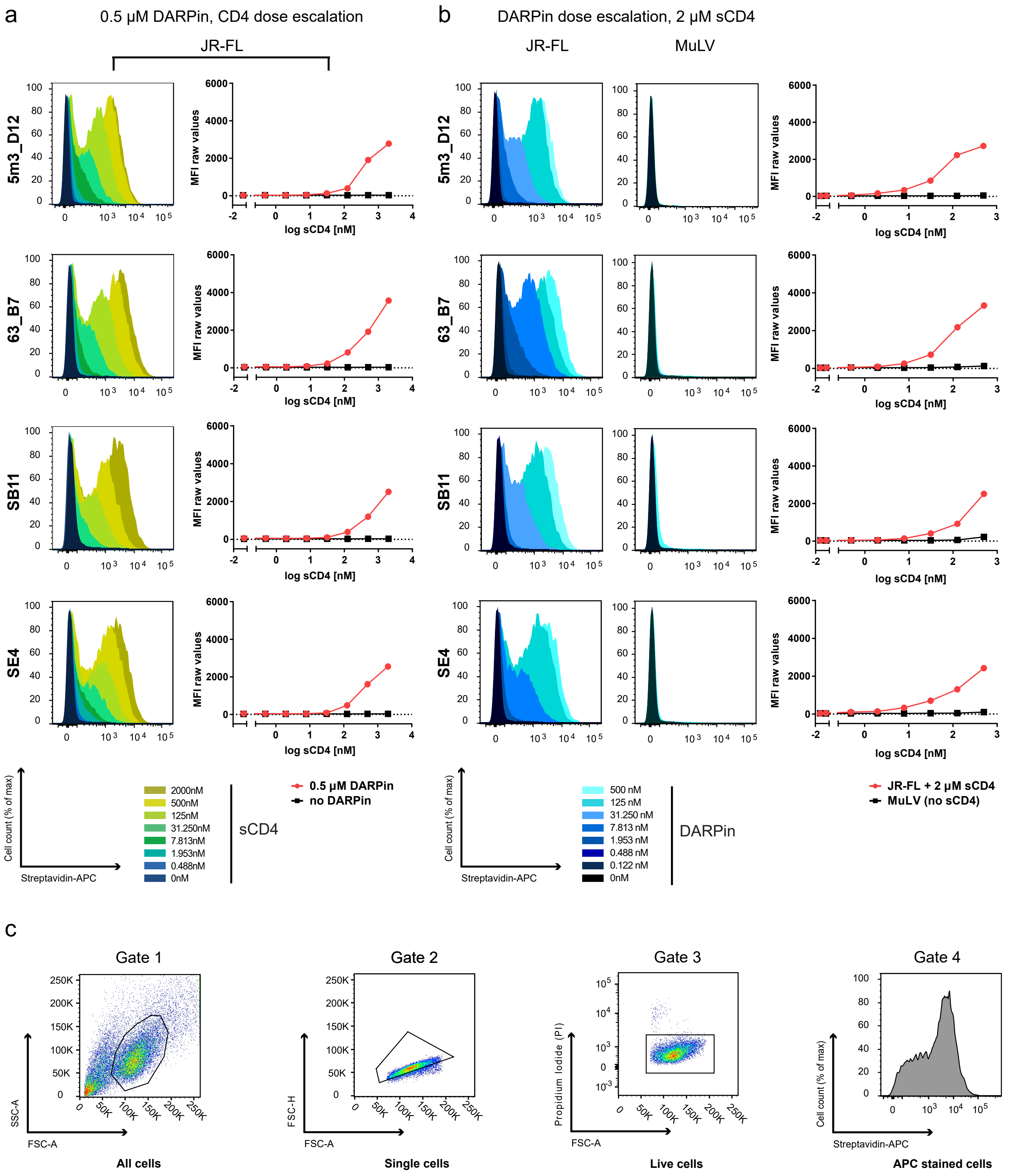

Supplementary Fig. 7: V3 DARPins depend on CD4 triggering to bind native HIV-1 Env.

Binding of biotinylated DARPins to cells expressing Env (JR-FL) was assessed by flow cytometry. **a** DARPin (0.5 µM) binding in the presence of increasing concentrations of sCD4. **b** DARPin titration in the presence of 2 µM sCD4. Mulv Env served as negative control. Histograms of normalized fluorescence intensities (left) and resulting plots of mean fluorescence intensity (MFI) versus concentration are depicted. **c** Gating strategy to obtain single, live cells from the pool of HEK 293 T cells harvested 36h post-transfection with Env-expression plasmid.

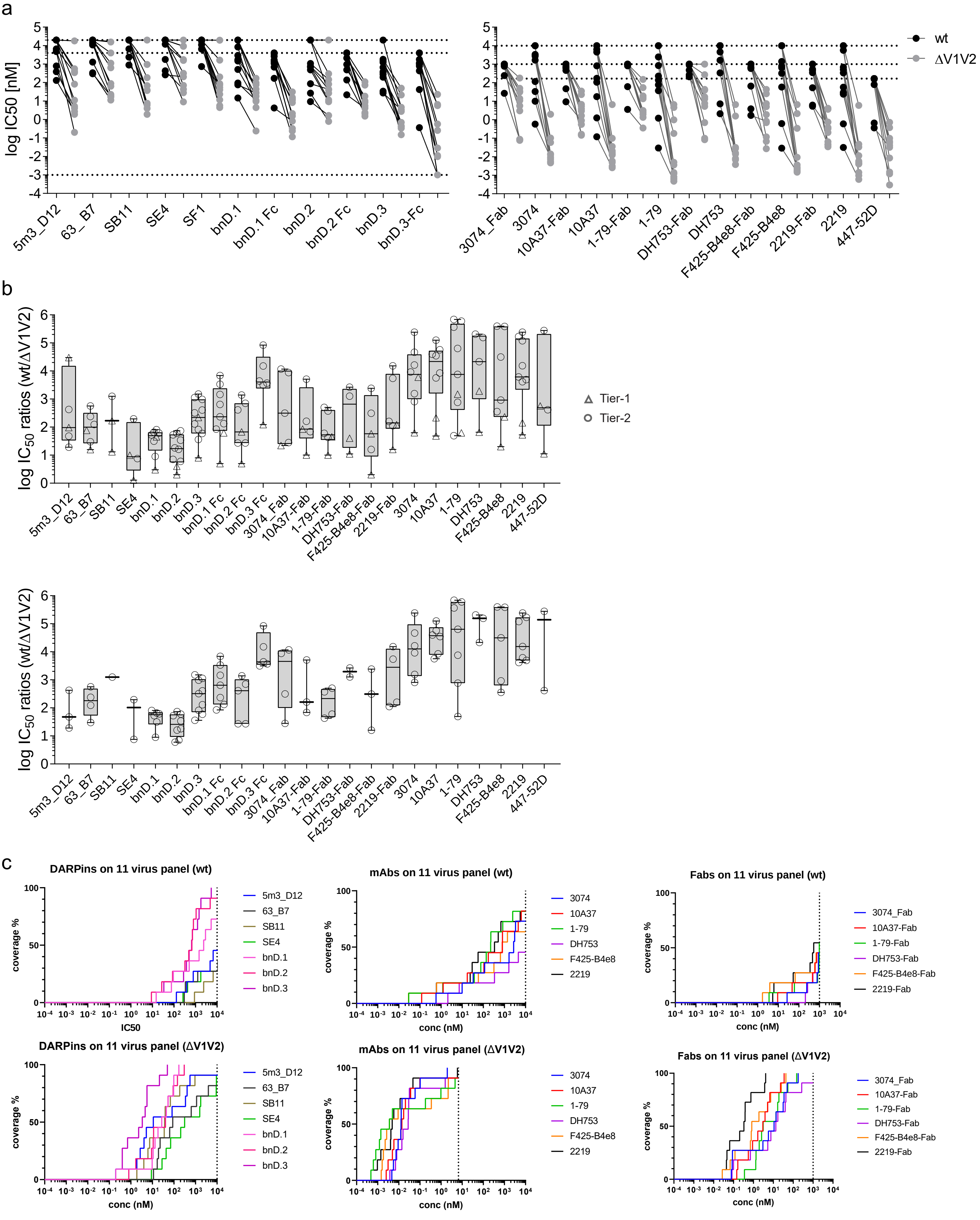

Supplementary Fig. 8: Protective effect of the V1V2 domain against V3-directed DARPins and antibodies Comparison of virus neutralization sensitivity (IC_{50}) with 11 wt and corresponding V1V2-deleted Envs (Supplementary Fig. 5b) to V3-directed DARPins, bivalent DARPin-Fc fusion proteins, Fabs and mAbs. Individual data points for wt and V1V2-deleted viruses represent geometric means from two independent experiments (n=2). Source data are provided as a Source Data file (see Supplementary Data Table 4). **a** IC_{50} values from corresponding wt and V1V2-deleted viruses are connected by a line. Minimum (only bnD.3-Fc: 0.002 nM) and maximum concentrations of inhibitors probed are indicated by dotted lines (20,000 nM for DARPins, 4,000 nM for bnD-Fc fusions (see also Fig. 5), 1,000 nM for Fabs, and 10,000 nM for mAbs except for 447-52D: 167 nM (=25 µg/ml)). bnD-Fcs and Fabs showed unspecific inhibition above the indicated maximum concentrations. **b** Box plots (center line: median; box limits extend from the 25th to 75th percentiles; whiskers indicate minimum and maximum values; all individual data points are shown) summarizing IC_{50} ratios (wt/ΔV1V2) calculated for all wt-ΔV1V2 pairs from **a**, excluding pairs with resistant strains. Low ratio values indicate low V1V2 shielding impact. Data including Tier-1 and Tier-2 Envs (top), data from Tier-2 Envs only (bottom). **c** Neutralization breadth in dependence of the inhibitor concentration on the 11 virus panel (either wt or V1V2-deleted versions) for DARPins, mAbs and Fabs respectively. Dotted lines indicate the maximum concentrations of inhibitors probed (as indicated in **a**).

a

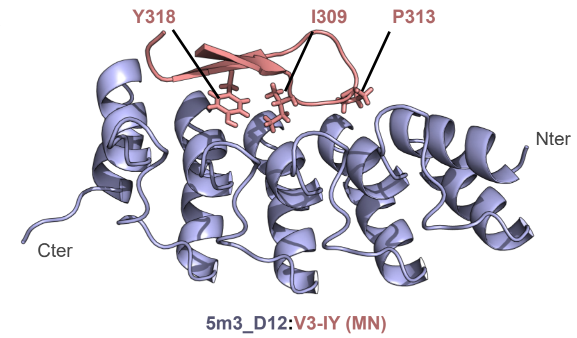

b

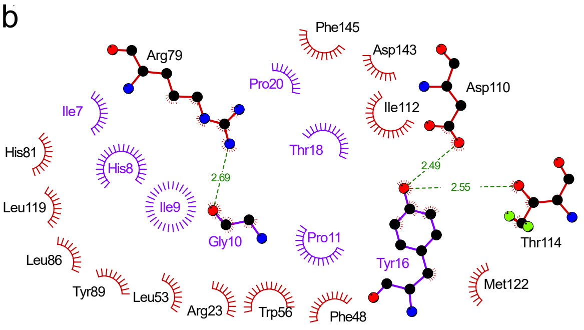

V3-IY (MN)	
HxB2 #	ligplot #
K305	K5
R306	R6
I307	I7
H308	H8
I309	I9
G312	G10
P313	P11
G314	G12
R315	R13
A316	A14
F317	F15
Y318	Y16
T319	T17
T320	T18
D-Pro	P19
L-Pro	P20

c

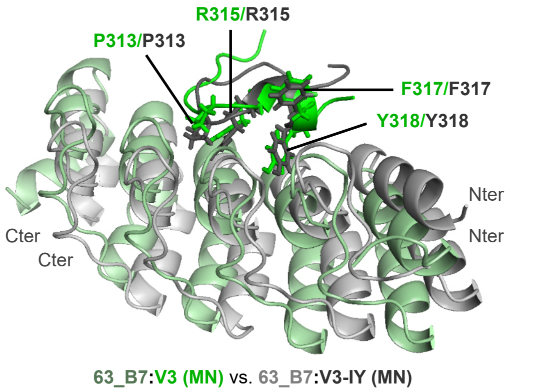

d

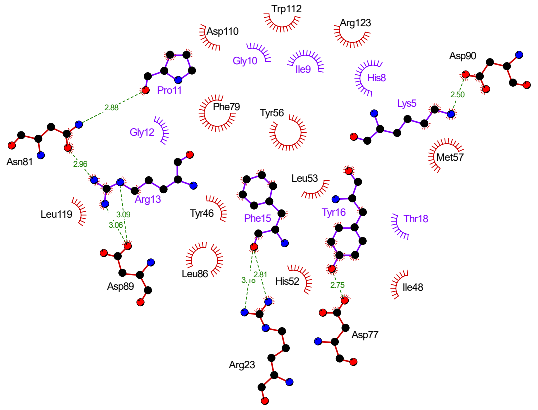

e

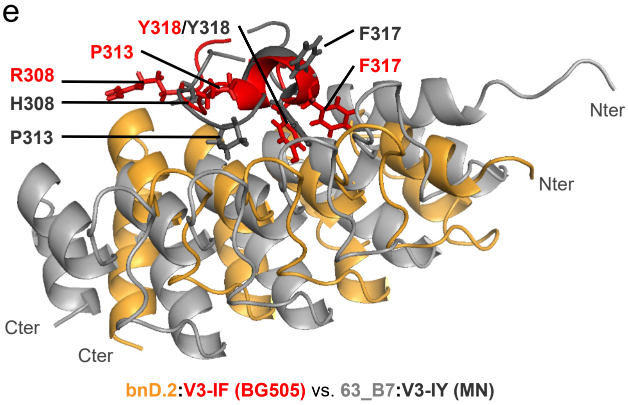

f

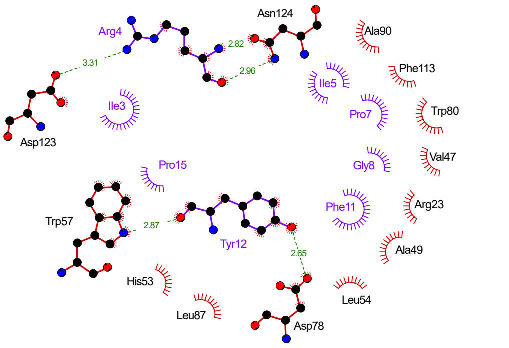

V3-IF (BG505)	
HxB2 #	ligplot #
K305	K1
S306	S2
I307	I3
R308	R4
I309	I5
G312	G6
P313	P7
G314	G8
Q315	Q9
A316	A10
F317	F11
Y318	Y12
A319	A13
D-Pro	P14
L-Pro	P15

g

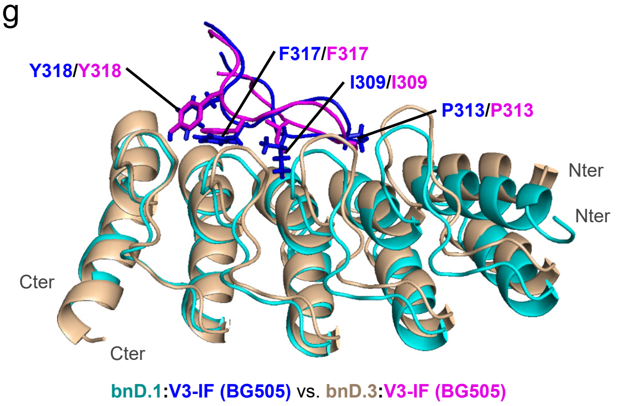

h

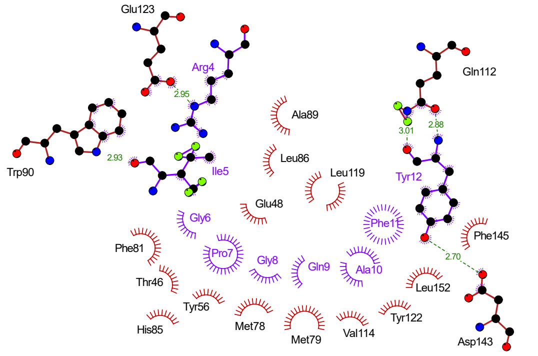

i

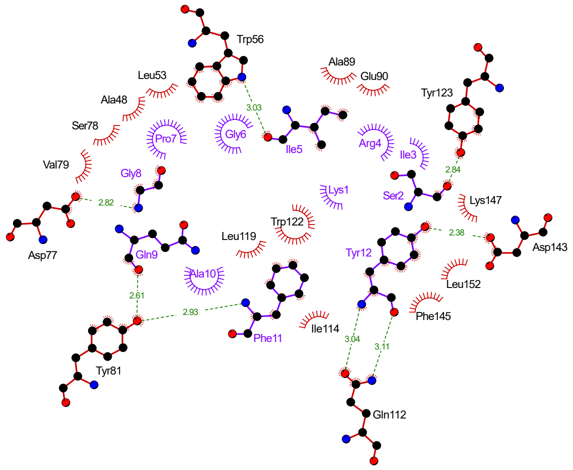

Supplementary Fig. 9: Crystal structures of DARPins in complex with V3 peptides

a Crystal structure of the complex between DARPin 5m3_D12 and mimetic V3-IY (MN). **b** Ligplot [112] corresponding to panel **a** (chains C and D of the asymmetric unit). Residue numbering in the ligplot is indicated in the legend. **c** Comparison of conformations adopted by a linear V3-crown and a structurally constrained V3-IY peptide (both derived from the MN strain) when crystallized in complex with DARPin 63_B7. The V3 peptides were aligned in PyMol. **d** Ligplot corresponding to the 63_B7:V3-IY (MN) complex in panel **c**. Residue numbering as in panel **b**. **e** Comparison of the bnD.2 and 63_B7 complexes by structural alignment of the V3 peptides in PyMol. **f** Ligplot corresponding to the bnD.2:V3-IF (BG505) complex in panel **e**. Residue numbering in the ligplot is indicated in a table. **g** Comparison of the bnD.1 and bnD.3 complexes by structural alignment of the V3 peptides in PyMol. **h** Ligplot corresponding to the bnD.1:V3-IF (BG505) complex in panel **g**. Residue numbering as in panel **f**. **i** Ligplot corresponding to the bnD.3:V3-IF (BG505) complex in panel **g**. Residue numbering as in panel **f**.

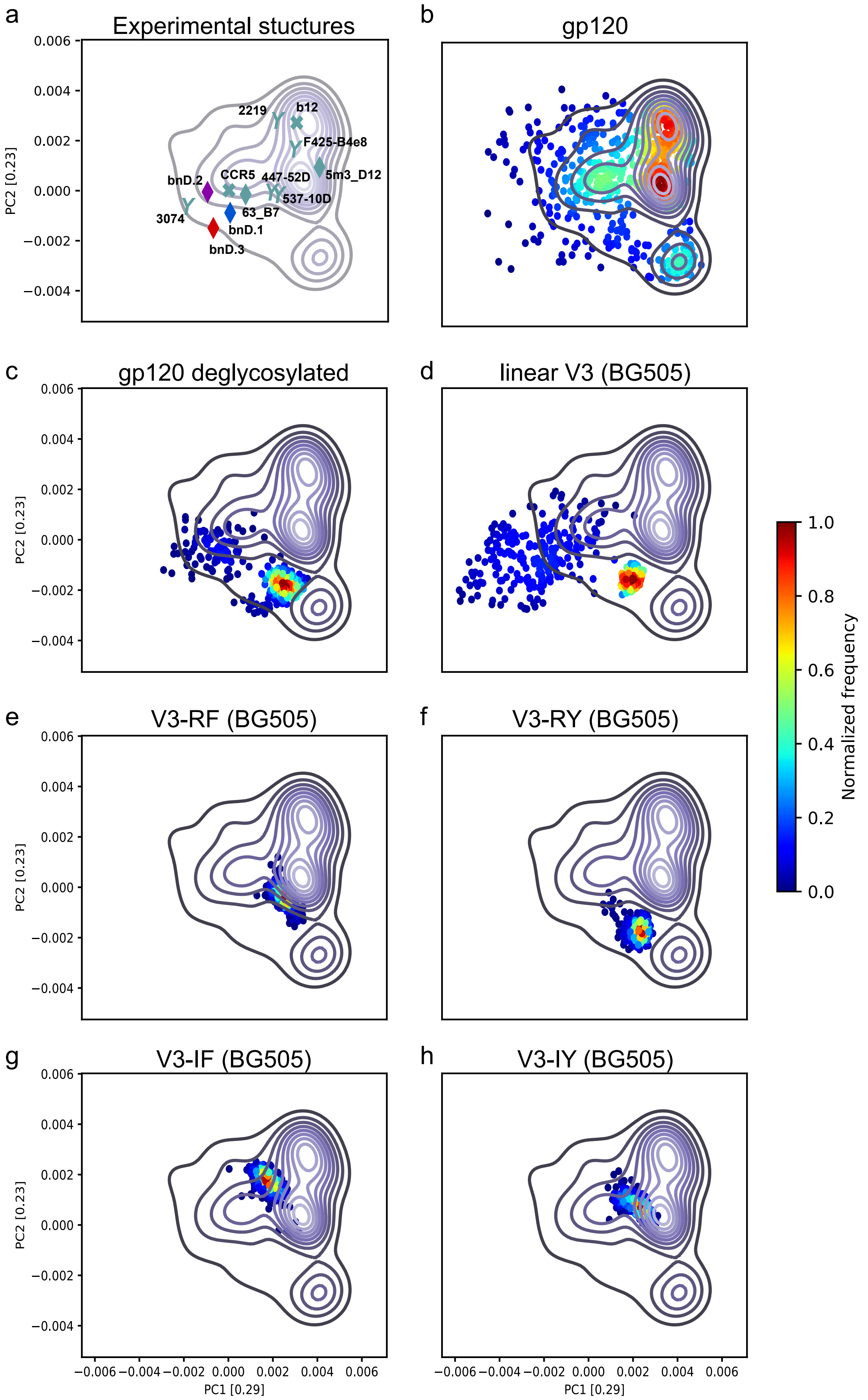

Supplementary Fig. 10: V3 crown molecular dynamics (MD) simulations comparing V3 on gp120 and V3 peptides.

A principal component analysis (PCA) was performed by considering the maximal common peptide length (residues 306-318) from the fully glycosylated gp120 bound to sCD4 MD simulation as a reference. The conformations sampled were projected into the eigenspace defined by the first two components. In each plot, the contour levels describe the frequency of conformations for V3 on glycosylated gp120 bound to sCD4 (MD initiated on the gp120 structure in complex with sCD4 and CCR5 (PDB ID: 6meo)) (dark blue to light blue gradient from low to high frequency). **a** The positions of experimentally determined liganded V3 structures in the conformational space are marked by a diamond, Y, or X in case the ligand is a V3 DARPin, a V3 mAb or else (CCR5 co-receptor or CD4-binding site mAb b12) and labeled with the name of the ligand. **b-h** For each construct indicated on top of the plot, the normalized frequency levels of individual V3 conformations derived by MD simulation are indicated by dots using a color gradient for comparison with the frequency of V3 conformations on glycosylated gp120 bound to sCD4 (contour levels).

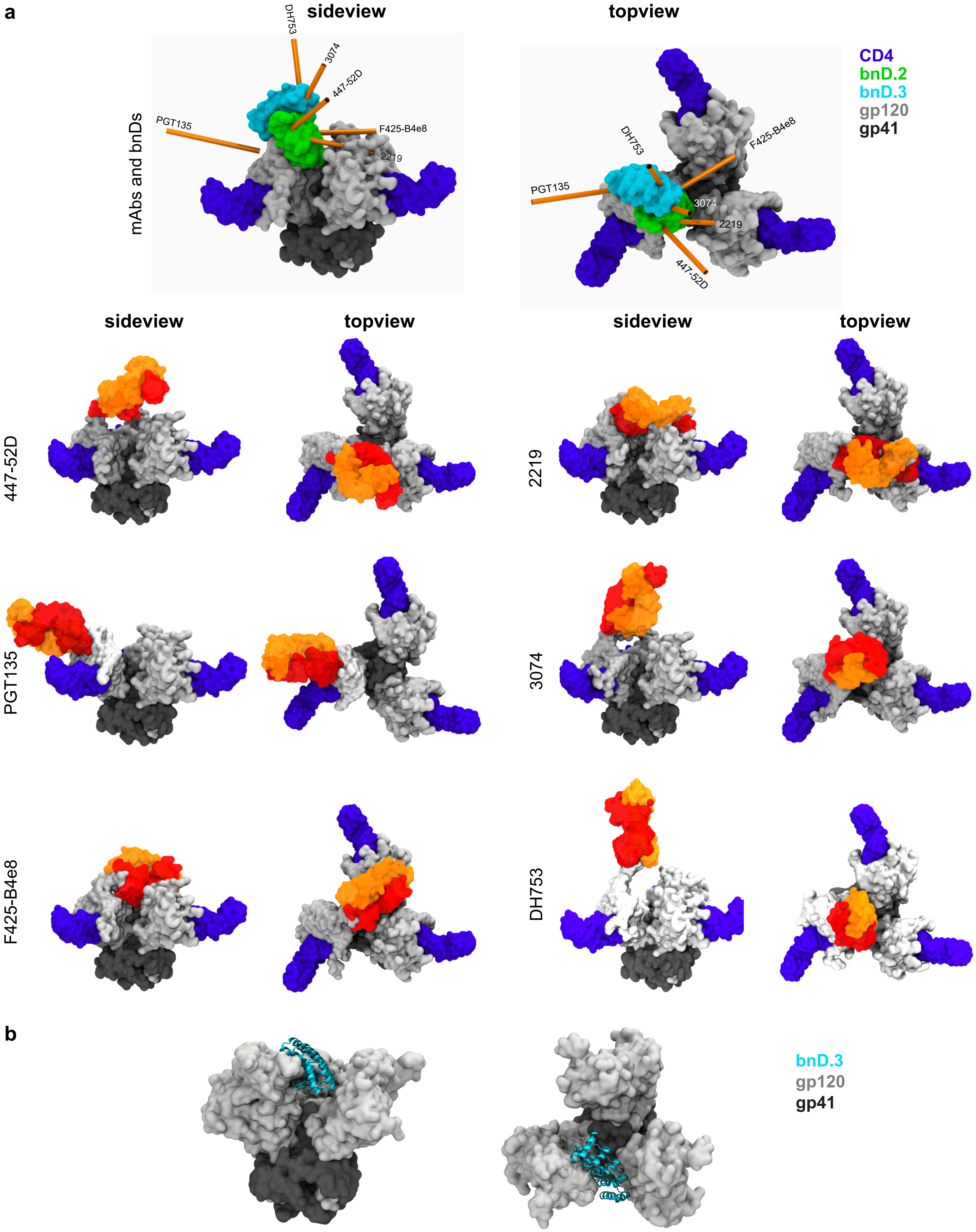

Supplementary Fig.11: Molecular modeling of Env trimer binding for V3-directed bnDs and mAbs

a Binding of V3-directed bnDs and mAbs modeled on the open Env trimer structure (bound by CD4 and the co-receptor mimicking mAb 17b, PDB ID: 5vn3 [11]. As in this complex V3 is not resolved, the V3 conformations derived by gp120 molecular dynamics simulations (Fig. 3c) were used for docking. V3 conformations that matched most closely the experimentally derived ligand-bound V3 structures (Fig. 3a) were used except for PGT135 which binds to the V3-base. Top: Docking is illustrated for all V3-directed agents together with mAb approach angles represented by orange rods. The approach angles of the mAbs were computed by a principal component analysis based on the structural coordinates of their respective Fab-fragments. Below the corresponding model complexes of the Fab fragments with the Env trimer are shown (with variable heavy chains in red, variable light chains in orange). **b** Molecular modeling of the interaction between bnD.3 and the partially open Env trimer (PDB ID: 5vn8 [11], the CD4-binding site mAb b12 of the original complex is not shown) illustrating the accessibility of the bnD.3-epitope.

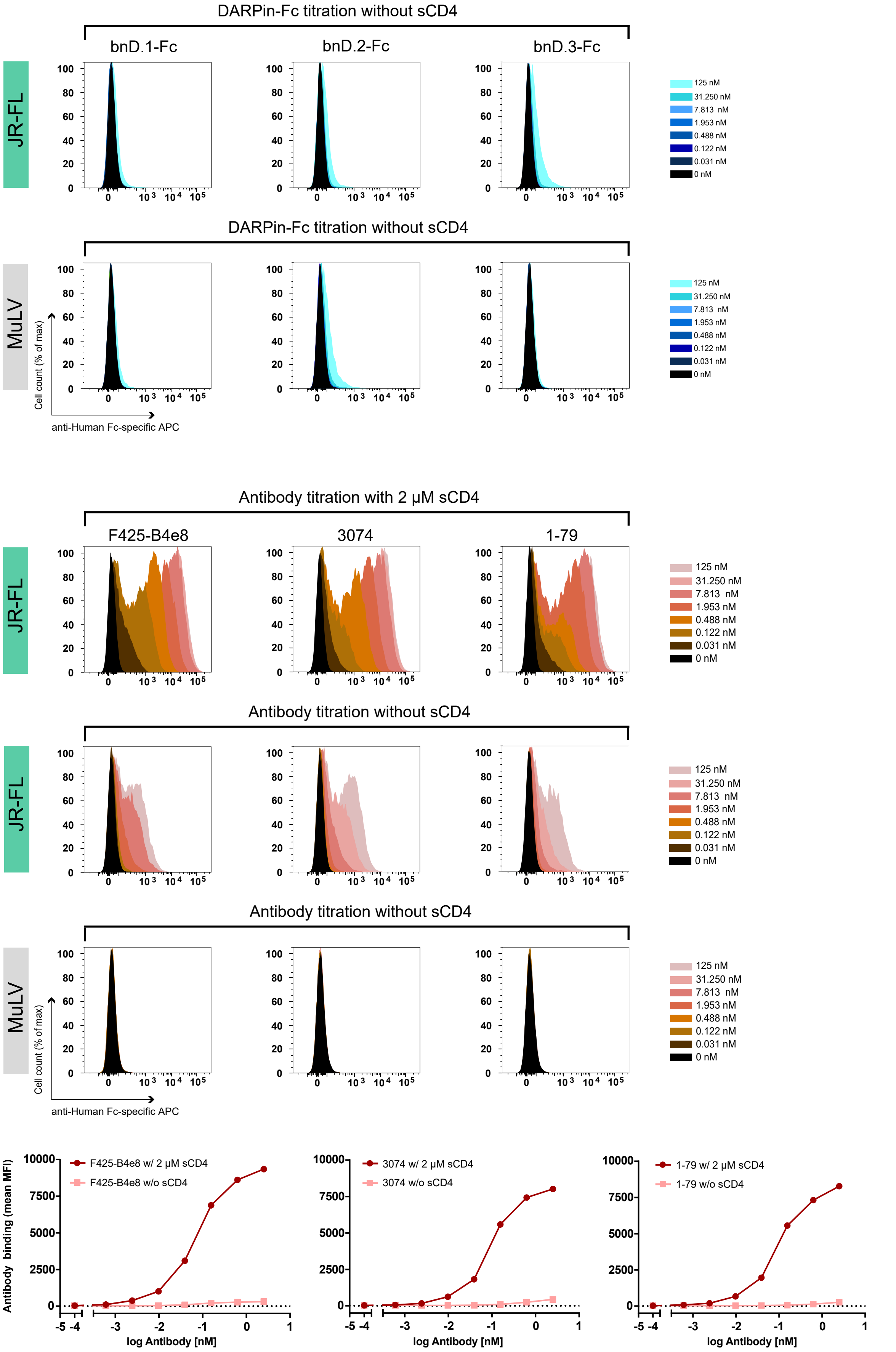

Supplementary Fig. 12: Bivalent DARPin-Fc fusions and mAbs targeting the V3-crown depend on CD4 triggering to efficiently bind native HIV-1 Env.

Binding of bivalent DARPin-Fc fusions and mAbs to cell surface expressed Env (JR-FL) measured by flow cytometry (see related data in Fig. 5b). V3-crown specific DARPin-Fc fusions and mAbs were titrated and incubated with cells in the presence or absence of 2 µM sCD4. Mulv Env served as negative control. Histograms of normalized fluorescence intensities (top panel) and dose-response curves showing mean fluorescence intensities (MFI, bottom panel) are depicted. The gating strategy is depicted in Supplementary Fig. 7c.

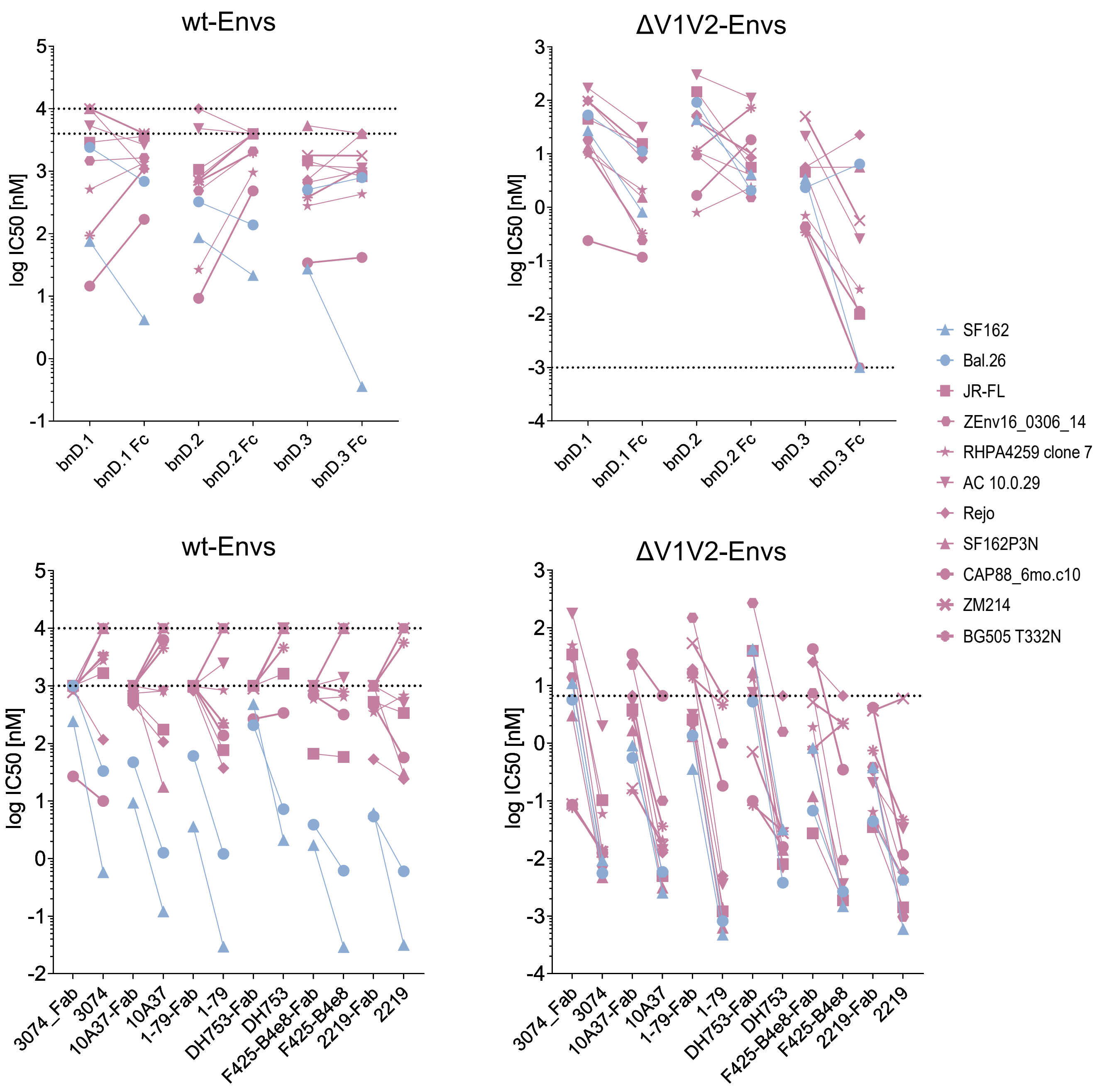

Supplementary Fig. 13: Effect of the V1V2 domain as well as inhibitor size and valency on neutralization

Comparison of virus neutralization sensitivity (IC_{50}) with 11 wt (left panels) and corresponding V1V2-deleted Envs (right panels) to mono- and bivalent V3-directed DARPins and mAbs. Tier-1 Envs are indicated in light blue, tier-2 Envs in pink. Pairs of bnDs/Fc-fused bnDs (upper panels) and Fabs/mAbs (lower panels) are connected by lines. IC_{50} values (geometric means of two independent experiments) are shown. Minimum (for bnD-Fc fusions only: 0.002nM) and maximum concentrations of inhibitors probed are indicated by dotted lines (10,000 nM for DARPins, 4,000 nM for DARPin-Fc fusions, 1,000 nM for Fabs; for mAbs different maximum concentrations were probed against viruses with wt (10,000 nM) and V1V2-deleted Envs (6,6 nM)). bnD-Fcs and Fabs showed unspecific inhibition above the indicated maximum concentrations. Source data are provided as Supplementary Data Table 4 in a Source Data file.

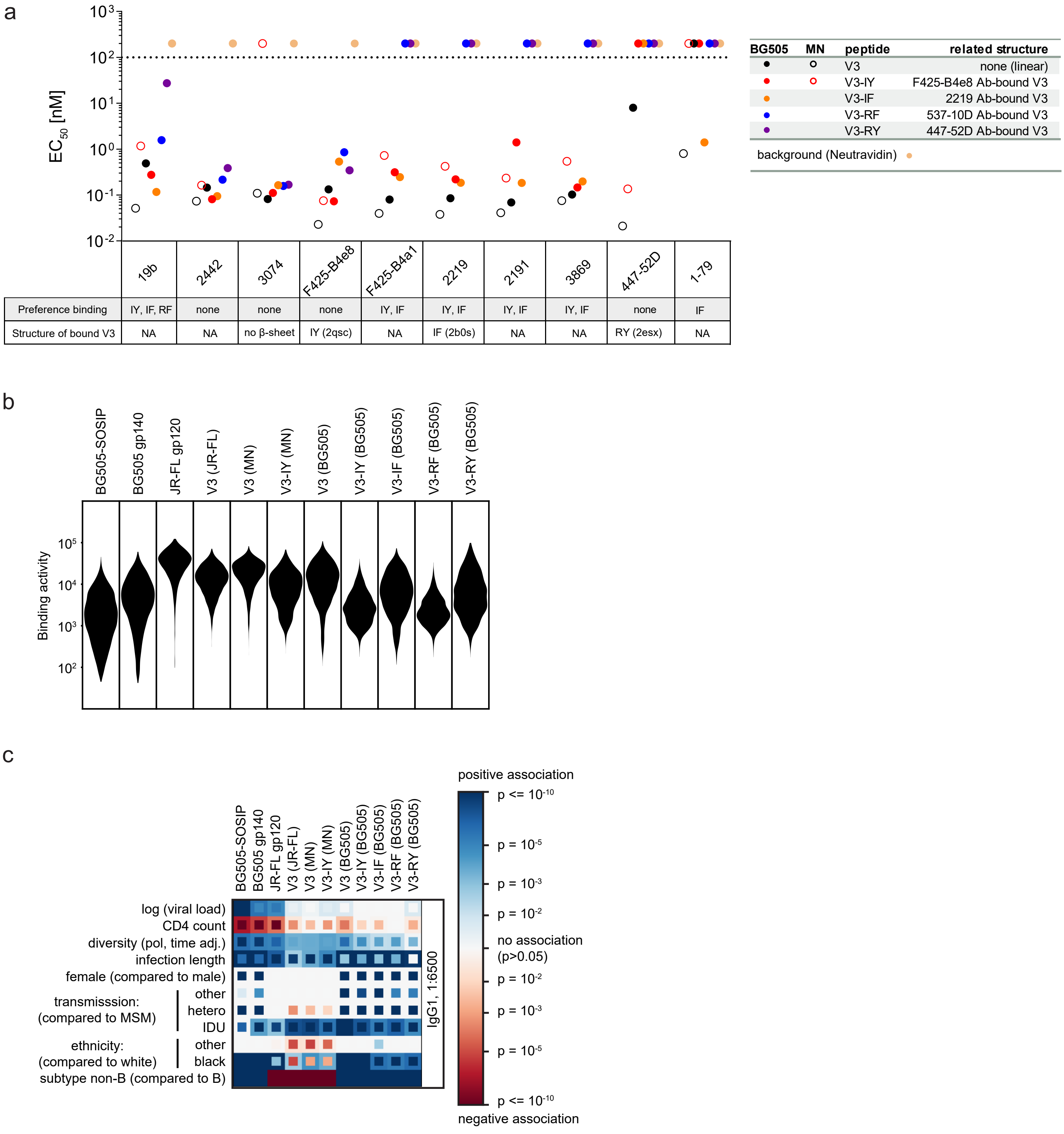

Supplementary Fig. 14: Assessing the V3 binding properties of the HIV-1 humoral immune response

a Deciphering structural V3 preferences of mAbs. Binding of V3-specific mAbs to biotinylated peptides immobilized to neutravidin-coated beads in a multiplexed Luminex assay. Linear V3 and cyclic structurally constrained V3-crown mimetics (V3-IY, V3-IF, V3-RF, V3-RY) are based on the V3 sequence from BG505 (clade A) or MN (clade B). Measured 50% effective concentrations (EC_{50}) are shown. The dotted line indicates the maximum mAb concentration probed. In case no EC_{50} below this concentration could be derived for a given peptide, the respective symbol is placed above this line. Neutravidin-coated beads were used as negative control. Preference binding: For each mAb the preferentially bound V3 peptide is indicated below. Structure of bound V3: for mAbs for which V3 bound structure data have been reported the adopted V3 register is depicted. NA (not available). **b** Summary of Swiss 4.5K Screen plasma survey (n=4,281) for V3 reactivity. Distribution of IgG1 absolute binding activities (mean fluorescence intensities (MFI)) at 1:6,500 plasma dilution against the indicated Env-derived targets (single measurements). Measurements on V3 crown mimetics were conducted in the current study, measurements on all other displayed Env antigens are reproduced from [13]. **c** Probing the influence of host, viral and disease parameters on IgG1 binding antibody responses. Data from the Swiss 4.5K Screen [13] cohort were used to probe which disease and host parameters influence the IgG1 response to the depicted HIV-1 antigens. Only data from n=3,159 patients without any missing/unknown parameters was included. Results from univariable (inner squares) and multivariable (outer squares, adjusted for multiple co-variates) linear regression analysis are depicted. Only significant associations (two sided p-value<0.05 from t-test; no adjustment for multiple testing) are colored. Color intensity represents the significance level of positive (blue) and negative (red) associations. Please note that, depending on the infecting subtype, a preference for certain antigens is expected (e. g. subtype B plasmas show higher binding to subtype B derived antigens (JR-FL and MN derived) whereas non-subtype plasmas bind BG505 antigens better). Source data are provided as Supplementary Data Tables 3 and 10 in Source Data files.

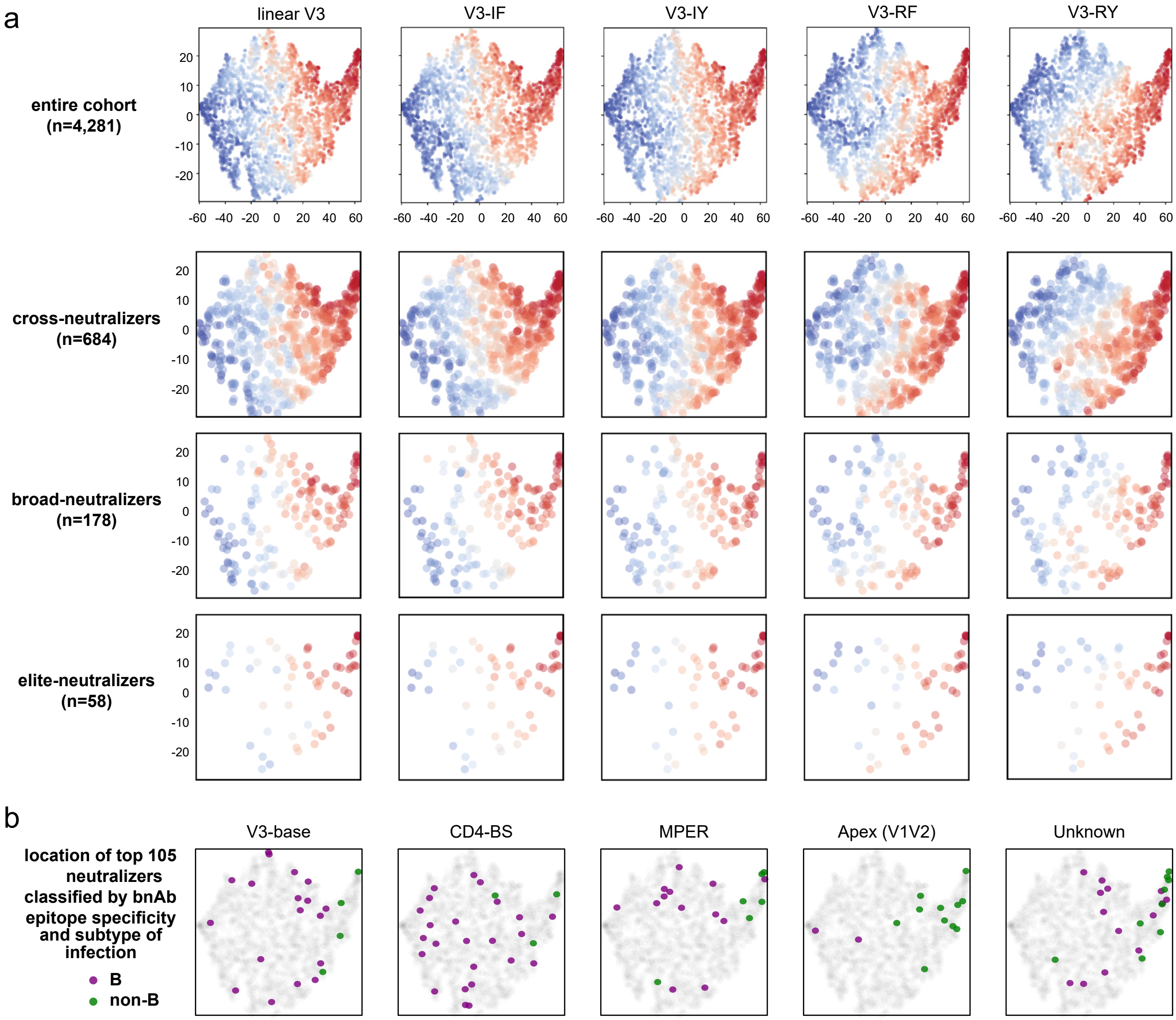

Supplementary Fig. 15: t-SNE analysis of IgG1 V3 peptides reactivity and neutralization breadth in the Swiss 4.5k cohort

a Top row: The same t-SNE map as shown in Fig. 6b (generated based on the relative IgG1 reactivities to the five V3 peptides for the n=4,281 Swiss 4.5K Screen participants) is colored based on relative IgG1 binding reactivities to linear V3 (BG505) and structurally constrained V3 mimetics (V3-IY, V3-IF, V3-RF, V3-RY). Red color denotes high binding activity and blue color denotes low binding activity to the V3 peptide indicated on the top of each panel. Rows 2-4 show the same map using the same color scheme but only show those plasma samples that exhibited cross (row 2), broad (row 3) and elite (row 4) neutralization capacity, as determined in [47]. **b** The top 105 neutralizing plasmas of the cohort were stratified by bnAb epitope specificity (using neutralization fingerprinting [47]) and were additionally classified according to the subtype of infection (B vs non-B). Their locations on the t-SNE plots from **a** are indicated by colored dots; all other plasma samples are shown in grey. Source data are provided as Supplementary Data Table 10 in a Source Data file.
