# Supplementary material for: Distinct conformations of the HIV-1 V3 loop crown are targetable for broad neutralization
Source: Nat Commun. 2021 Nov 18;12:6705. doi: 10.1038/s41467-021-27075-0 (PMC8602657; doi:10.1038/s41467-021-27075-0)
Supplement: Supplementary file 2 — Description of Additional Supplementary Files [file 41467_2021_27075_MOESM2_ESM.pdf]

Description of Additional Supplementary Files

File Name: Supplementary Data 1
Description: Neutralization breadth and potency of V3 directed DARPins and antibodies

File Name: Supplementary Data 2
Description: Neutralization fingerprint analysis of V3-Crown bnDs and bnAbs

File Name: Supplementary Data 3
Description: Env binding properties of V3-crown DARPins and V3 specific antibodies

File Name: Supplementary Data 4
Description: Protective effect of the V1V2 domain against V3-directed DARPins and antibodies

File Name: Supplementary Data 5
Description: Data collection and structure refinement statistics of DARPin:V3 co-crystal structures

File Name: Supplementary Data 6
Description: Distribution of the buried surface area (BSA) on V3 in DARPin:V3 co-crystal structures

File Name: Supplementary Data 7
Description: Envelope mutational scanning identifies key residues that determine sensitivity to V3 bnDs.

File Name: Supplementary Data 8
Description: Repetitive testing of JR-CSF Env alanine point mutants in a pseudovirus inhibition assay for identification of DARPin resistance mutations.

File Name: Supplementary Data 9
Description: Effect of size and valency on V3-crown bnD Env binding and neutralization activity

File Name: Supplementary Data 10
Description: All raw data related to Figure 6 and Supplementary Figures 14 and 15

File Name: Supplementary Data 11
Description: Overview of results from the linear regression analysis presented in Figure 6c and Supplementary Figure 14c

File Name: Supplementary Data 12
Description: Sources and references for antibodies and recombinant proteins used in the study
