# Supplementary material for: Distinct conformations of the HIV-1 V3 loop crown are targetable for broad neutralization
Source: Nat Commun. 2021 Nov 18;12:6705. doi: 10.1038/s41467-021-27075-0 (PMC8602657; doi:10.1038/s41467-021-27075-0)
Supplement: Supplementary file 15 — Reporting Summary [file 41467_2021_27075_MOESM15_ESM.pdf]

nature research

Corresponding author(s): Alexandra Trkola

Last updated by author(s): Oct 27, 2021

Reporting Summary

Nature Research wishes to improve the reproducibility of the work that we publish. This form provides structure for consistency and transparency in reporting. For further information on Nature Research policies, see our Editorial Policies and the Editorial Policy Checklist.

Statistics

For all statistical analyses, confirm that the following items are present in the figure legend, table legend, main text, or Methods section.

n/a	Confirmed	
☐	☒	The exact sample size (*n*) for each experimental group/condition, given as a discrete number and unit of measurement
☐	☒	A statement on whether measurements were taken from distinct samples or whether the same sample was measured repeatedly
☐	☒	The statistical test(s) used AND whether they are one- or two-sided<br>*Only common tests should be described solely by name; describe more complex techniques in the Methods section.*
☐	☒	A description of all covariates tested
☐	☒	A description of any assumptions or corrections, such as tests of normality and adjustment for multiple comparisons
☐	☒	A full description of the statistical parameters including central tendency (e.g. means) or other basic estimates (e.g. regression coefficient) AND variation (e.g. standard deviation) or associated estimates of uncertainty (e.g. confidence intervals)
☐	☒	For null hypothesis testing, the test statistic (e.g. *F*, *t*, *r*) with confidence intervals, effect sizes, degrees of freedom and *P* value noted<br>*Give P values as exact values whenever suitable.*
☒	☐	For Bayesian analysis, information on the choice of priors and Markov chain Monte Carlo settings
☒	☐	For hierarchical and complex designs, identification of the appropriate level for tests and full reporting of outcomes
☒	☐	Estimates of effect sizes (e.g. Cohen's *d*, Pearson's *r*), indicating how they were calculated

Our web collection on statistics for biologists contains articles on many of the points above.

Software and code

Policy information about availability of computer code

Data collection	Glycan modelling was performed using python package Glycosylator 1.0. Subsequent molecular dynamics simulations were carried out using ACEMD 3.0 software as described in the Materials and Methods section. The RosettaDock (2018.33.60351) procedure was used for the protein-protein docking.
Data analysis	All data analyses are detailed in the Materials and Methods section. Binding and neutralization data was analysed using GraphPad PRISM7 or 9. Flow cytometry data was analyzed using FlowJo 10 software. X-ray diffraction data of protein crystals was processed using XDS, XSCALE and XDSCONV (Version October 15, 2015). Initial phases were obtained by molecular replacement using PHASER (2.6.0). Structure refinement was done using REFMAC5 (version 5.8.0135), BUSTER (version 2.10.1), Phenix-Refine (version 1.11_2567) and COOT (version 0.8.8). Binding interfaces were analyzed with LigPlot+ (v1.4.2) and QtPISA (version 2.0.4). Figures of structures were prepared with PyMOL version 1.8. Statistical analyses were performed in Python 2.7 or 3.7 (as indicated) using the packages scipy.stats, statsmodels, and tsne.

For manuscripts utilizing custom algorithms or software that are central to the research but not yet described in published literature, software must be made available to editors and reviewers. We strongly encourage code deposition in a community repository (e.g. GitHub). See the Nature Research guidelines for submitting code & software for further information.

Data

Policy information about availability of data

All manuscripts must include a data availability statement. This statement should provide the following information, where applicable:

- Accession codes, unique identifiers, or web links for publicly available datasets
- A list of figures that have associated raw data
- A description of any restrictions on data availability

The structural data on DARPin:V3 complexes generated in this study (Fig. 3 and Supplementary Fig. 9) have been deposited in the Protein Data Bank (PDB) database

under accession codes 7DNE (5m3_D12:V3-IY), 7DNF (63_B7:V3-IY), 7DNG (63_B7:V3), 7B4T (bnD.1:V3-IF), 7B4U J06 and 7B4V J32 (bnD.2:V3-IF) and 7B4W (bnD.3:V3-IF). Supplementary Table 5 with corresponding data collection and refinement statistics is included in the source data file. Other publicly available datasets from the PDB used in this study (Figures 3 and 4, Supplementary Figs 3, 10, 11, and 14) are accessible under PDB IDs 6MEO (CCR5:gp120:sCD4), 5VN8 (b12 Fab:B41-SOSIP trimer), 3GHE (537-10D Fab:V3), 2QSC (F425-B4e8 Fab:V3), 2B0s (2219 Fab:V3), 3MLX (3074 Fab:V3), 4M1D and 2ESX (447-52D Fab:V3), 6MNR (DH753 Fab:V3), 4JM2 (PGT135 Fab:gp120:17b Fab:sCD4).

All Supplementary Tables are provided as source data with this paper. Additional source data related to Rusert et al. (Nat. Medecine, 2016) and Kadelka et al. (J. Exp. Med., 2018), can be found online under https://doi.org/10.1038/nm.4187 and https://doi.org/10.1084/jem.20180246, respectively.

Field-specific reporting

Please select the one below that is the best fit for your research. If you are not sure, read the appropriate sections before making your selection.

- [x] Life sciences
- [ ] Behavioural & social sciences
- [ ] Ecological, evolutionary & environmental sciences

For a reference copy of the document with all sections, see nature.com/documents/nr-reporting-summary-flat.pdf

Life sciences study design

All studies must disclose on these points even when the disclosure is negative.

Sample size

The number of samples from HIV infected individuals is predetermined through the size of the Swiss HIV-1 Cohort (SHCS) and the Zurich Primary HIV Infection Study (ZPHI) cohort as well as selection criteria applied for the Swiss 4.5K Screen (Rusert et al., Nat. Medecine, 2016).

Data exclusions

No data was excluded.

Replication

In the luminex-based binding assay of IgG in human plasma to HIV-1 antigens (Figure 6, Supplementary Figures 14 and 15), monoclonal HIV-1–specific antibodies (PG09, 2F5, 19b, 2G12, 447-52D, and HIV IG) were analyzed with each experiment as assay quality control (see also Kadelka et al., 2018). The accuracy of the test allowed that due to the large number of samples (4’281) and the limited availability of these historic clinical only single measurements without replicates were done as specified in (Kadelka et al., 2018).

For all other experiments, all attempts at replication with optimized parameters as detailed in the materials and methods section were successful: DARPin binding ELISA (Fig. 1a and Supplementary Figure 3c: n=3) , Pseudovirus inhibition assays (Figure 1: n=1-5 depending on the virus), (Figures 5d, Supplementary Figures 8 and 13: n=2). For binding ELISA with bnD-Fcs (Figure 5c), competition binding ELISA (Figure 2b and Supplementary Figure 3d) and cell-surface binding experiments using flow-cytometry (Figures 2a, 5b and Supplementary Figures 7 and 12), representative data from at least two similarly conducted experiments are shown.

Neutralization screens with a JR-CSF Env mutant panel: 128 JR-CSF mutant pseudoviruses carrying gp120 point mutations (Figure 4, Supplementary Tables 7 and 8) were probed for sensitivity to DARPins and selected mAbs in a two-step screening approach to detect resistance conferring mutants. In a first run, all mutant viruses of the JR-CSF Env panel were screened against all inhibitors. Mutants that passed in this first screen — a pre-set threshold of resistance (5-fold over IC50 against wt JR-CSF) against one DARPin — were followed up and retested against all DARPins, yielding in total at least two independent tests (Supplementary Table 8). Mutants that showed no effect against any of the inhibitors were not followed up and hence only tested once.

Randomization

Acquisition of the plasma IgG binding data to a range of HIV-derived targets by a customized Luminex-assay and allocation of samples/participants into groups during data analysis was conducted as described in Kadelka et al., J. Exp. Med., 2018.

Blinding

Personnel who conducted the HIV-1–binding antibody analysis reported here and in Kadelka et al., 2018 and the previously reported neutralization screen (Rusert et al., 2016) were blinded, i.e. had no information on patient demographics, neutralization activity and group allocation at the time of data collection.

Reporting for specific materials, systems and methods

We require information from authors about some types of materials, experimental systems and methods used in many studies. Here, indicate whether each material, system or method listed is relevant to your study. If you are not sure if a list item applies to your research, read the appropriate section before selecting a response.

Materials & experimental systems

n/a	Involved in the study	
☐	☒	Antibodies
☐	☒	Eukaryotic cell lines
☒	☐	Palaeontology and archaeology
☒	☐	Animals and other organisms
☐	☒	Human research participants
☐	☒	Clinical data
☒	☐	Dual use research of concern

Methods

n/a	Involved in the study	
☒	☐	ChIP-seq
☐	☒	Flow cytometry
☒	☐	MRI-based neuroimaging

Antibodies

Antibodies used	- mouse anti-FLAG® antibody (Sigma Aldrich, clone M2, Cat#F1804 and Cat#F3165);<br>- alkaline phosphatase-conjugated polyclonal goat anti-mouse IgG (whole molecule) secondary antibody (Sigma Aldrich, Cat#A3562);<br>- monoclonal alkaline phosphatase conjugated mouse anti-polyhistidin antibody (Qiagen, clone HIS-1, Cat#A5588);<br>- phycoerythrin (PE)-labeled secondary antibody specific to isotype IgG1 (Southern Biotech, Cat#9054-09, clone HP6001);<br>- allophycocyanin (APC) conjugated goat anti-Human IgG $F(ab')_2$ secondary antibody (Jackson ImmunoResearch Europe, Ely, UK; Cat#109-136-170);<br>- polyclonal goat anti-human IgG (Fc specific) alkaline phosphatase-conjugated secondary antibody (Sigma-Aldrich, Cat#A9544, Lot. 048K4821);<br>- References and sources for all HIV-1 Envelope protein specific antibodies used in this study are detailed in Supplementary Table 11. For antibodies obtained through the National Institutes of Health AIDS Reagent Program (NIH ARP), the catalog numbers are specified.
Validation	Mouse anti-FLAG® IgG1 antibody (Sigma Aldrich, clone M2, Cat#F1804 and Cat#F3165) was validated by the company for ELISA, Immunblotting, Immunprecipitation, Immunohistochemistry, Immuncytochemistry, and Immunfluorescence-Assays and has been optimized for detection of FLAG-tagged proteins in mammalian, plant and bacterial expression systems. The M2 antibody is not Calcium dependent (unlike clone M1) and is able to recognized the FLAG-tag at the N-terminus, C-terminus and at internal sites.<br><br>Alkaline phosphatase conjugated mouse anti-polyhistidin IgG2a antibody (Qiagen, clone HIS-1, Cat#A5588) was validated by the company for ELISA, Immunblotting and Dotblot-Assays. Anti-polyHistidine-Alkaline Phosphatase recognizes native as well as denatured / reduced forms of synthetic polyHistidine or polyHistidine-tagged fusion proteins. The product is reactive with fusion protein expressed by prokaryotic expression vectors and may be useful in various immunotechniques to identify the expression of a polyHistidine fusion protein in bacteria, bacterial lysates or cells and tissues transfected with a polyHistidine-tagged fusion protein expressing vectors. Several publications cite use of this product in their studies (Dong, S. et al., Science, 343(6170), 552-555 (2014) and Yang, M. et al., Nat. Chem. Biol., 14(12), 1109-1117 (2018)).<br><br>All HIV-1 Envelope protein specific antibodies were validated in the references provided in Supplementary Table 11 and their specificity was verified by the use of appropriate background controls in the current study.

Eukaryotic cell lines

Policy information about cell lines

Cell line source(s)	HEK 293-T cells were obtained from the American Type Culture Collection and TZM-bl cells through the NIH AIDS Reagent Program. HEK 293T FreestyleTM suspension (293F and Expi293F) cells were purchased from Thermo Fisher.
Authentication	None of the cell lines used were authenticated again after reception from the specified original source.
Mycoplasma contamination	In the Trkola laboratory, cell lines are routinely tested for mycoplasma contamination. No such contamination was detected in the cells used for the present study.
Commonly misidentified lines (See ICLAC register)	No commonly misidentified cell lines were used in the study.

Human research participants

Policy information about studies involving human research participants

Population characteristics	The SHCS, founded in 1988, is highly representative of the HIV epidemiology in Switzerland as it includes an estimated 53% of all HIV cases diagnosed in Switzerland since the onset of the epidemic, 72% of all patients receiving ART in Switzerland, and 69% of the nationwide registered AIDS cases.<br>The Zurich Primary HIV Infection study (ZPHI) specifically enrolls patients with documented acute or recent primary HIV-1 infection.<br>The following eight host, viral, and disease parameters were included in the analysis of the current study: viral load, CD4 count, infection length, virus diversity, gender, transmission mode, ethnicity, and viral pol subtype. These covariate-relevant population characteristics are provided as Supplementary Table 10 in the Source data file. The distributions of the parameters across the 4,281 patients are summarized in Rusert et al. (Nat. Medecine, 2016) and Kadelka et al. (J. Exp. Med., 2018), Table S2.
Recruitment	The Swiss HIV Cohort Study (SHCS) is a prospective, nationwide, longitudinal, non-interventional, observational, clinic-based cohort with semi-annual visits and blood collections, enrolling all HIV-infected adults living in Switzerland.<br>The Zurich Primary HIV Infection study (ZPHI) is an ongoing, observational, non-randomized, single center cohort founded in 2002.
Ethics oversight	The SHCS and the ZPHI have been approved by the ethics committee of the participating institutions (Kantonale Ethikkommission Bern, Ethikkommission des Kantons St. Gallen, Comité départemental d'éthique des spécialités médicales et de médicine communautaire et de premier recours, Kantonale Ethikkommission Zürich, Repubblica e Cantone Ticino - Comitato Ethico Cantonale, Commission cantonale d'éthique de la recherche sur l'être humain, Ethikkommission beider Basel for the SHCS and Kantonale Ethikkommission Zürich for the ZPHI) and written informed consent had been obtained from all participants.

Note that full information on the approval of the study protocol must also be provided in the manuscript.

Clinical data

Policy information about clinical studies

All manuscripts should comply with the ICMJEguidelines for publication of clinical research and a completedCONSORT checklist must be included with all submissions.

Clinical trial registration	The SHCS is registered under the Swiss National Science longitudinal platform: http://www.snf.ch/en/funding/programmes/longitudinal-studies/Pages/default.aspx#Currently%20supported%20longitudinal%20studies.<br>The Zurich Primary HIV Infection study (ZPHI) is registered on ClinicalTrials.gov with the ID NCT00537966.
Study protocol	Detailed information on the SHCS and ZPHI cohorts is openly available on http://www.shcs.ch and http://www.ClinicalTrials.gov respectively.
Data collection	The Swiss HIV Cohort Study (SHCS) is a prospective, nationwide, longitudinal, non-interventional, observational, clinic-based cohort with semi-annual visits and blood collections, enrolling all HIV-infected adults living in Switzerland. ZPHI study is a is an open label, non-randomized, observational, single center study at the University Hospital Zurich, Division of Infectious Diseases and Hospital Epidemiology. This study started in January 2002 and is estimated to be completed in January 2025.<br>Both SHCS and ZPHI maintain a comprehensive, longitudinal, anonymous data collection of all participants, including extensive clinical and demographic data. The following data were used in the current study: longitudinal viral load and CD4 measurements, clinical history (antiretroviral drug history, infection length, and patient demographics), and pol nucleotide sequence data from genotypic antiretroviral drug resistance tests. The ethnicity (race) information is self-reported by the SHCS and ZPHI study participants at enrollment. Study nurses or study physicians use a structured interview questionnaire and participants are asked whether they belong to one of the following races/ethnicities: white, black, Hispano-American, or Asian. These categories cover the major ethnicities in Switzerland. Health care access is guaranteed for all races/ethnicities living in Switzerland and the same is true for participation in the SHCS and the ZPHI. Please see the supplementary note of Rusert et al. (2016) for further details on how patient data were recorded.<br>The following eight host, viral, and disease parameters were included in the analysis of the current study: viral load, CD4 count, infection length, virus diversity, gender, transmission mode, ethnicity, and viral pol subtype. These covariate-relevant population characteristics are provided as Supplementary Table 10 in the Source data file. The distributions of the parameters across the 4,281 patients are summarized in Rusert et al. (Nat. Medecine, 2016) and Kadelka et al. (J. Exp. Med., 2018), Table S2.
Outcomes	The primary outcome measure of the ZPHI is to evaluate the effect of early-cART on the viral setpoint, which however is not relevant to the present study.<br>Here neutralization breadth was used as a categorical response variable. Patients were defined to have neutralization breadth if their plasma reached cross-, broad- or elite-neutralization activity score as determined in Rusert et al., Nat. Med., 2016 and Kouyos et al., Nature, 2018. Patients with no or weak neutralization activity scores were categorized as having no neutralization.<br>Viral load and CD4 level (both measured at time of sampling), viral pol diversity and infection length were included as continuous variables. The remaining four factors were used as categorical variables and analyzed in relation to the reference category (sex: reference male; mode of transmission: reference men having sex with men (MSM); ethnicity: reference White; HIV-1 clade: reference clade B, neutralization breadth: reference no neutralization).

Flow Cytometry

Plots

Confirm that:

- [x] The axis labels state the marker and fluorochrome used (e.g. CD4-FITC).
- [x] The axis scales are clearly visible. Include numbers along axes only for bottom left plot of group (a 'group' is an analysis of identical markers).
- [x] All plots are contour plots with outliers or pseudocolor plots.
- [x] A numerical value for number of cells or percentage (with statistics) is provided.

Methodology

Sample preparation	HEK 293-T cells (obtained from the American Type Culture Collection) were co-transfected with the desired Env-expression plasmid and the pCMV-rev expression helper plasmid in 4:1 ratio. After 36h, cells were incubated with biotinylated DARPins in the presence or absence of sCD4-183 at the indicated concentrations for 20 minutes at RT. Bound DARPins were detected via APC-Cy7 conjugated streptavidin (BD Biosciences, San Jose, USA; Cat#554063), bound DARPin-Fc fusions and mAbs via allophycocyanin (APC) conjugated goat anti-Human IgG F(ab')$_2$ (Jackson ImmunoResearch Europe, Ely, UK; Cat#109-136-170)
Instrument	FACSVerse system (BD Biosciences)
Software	FlowJo 10 software (FlowJo LLC, Ashland, USA)
Cell population abundance	No post-sort fractions of live single cells were analyzed.
Gating strategy	Transfected HEK 293T cells were gated by FSC/SSC for single cells (eliminating cell debris), PI staining allowed gating for the live single cell fraction which was analyzed for APC-positive cells (Mulv-Envelope transfected cells served as negative control). The gating strategy is exemplified in Supplementary Figure 7c.

- [x] Tick this box to confirm that a figure exemplifying the gating strategy is provided in the Supplementary Information.
